# The influence of rat strain on the development of neuropathic pain and comorbid anxio-depressive behaviour after nerve injury

**DOI:** 10.1038/s41598-020-77640-8

**Published:** 2020-12-01

**Authors:** Sara Hestehave, Klas S. P. Abelson, Tina Brønnum Pedersen, David P. Finn, Daniel R. Andersson, Gordon Munro

**Affiliations:** 1 H. Lundbeck A/S, Valby, Denmark; 2 Department of Experimental Medicine, Faculty of Health and Medical Sciences, University of Copenhagen, Copenhagen, Denmark; 3 Department of Cell and Developmental Biology, University College London, Medawar Building, Malet Place, London, WC1E 6BT UK; 4 Pharmacology and Therapeutics, School of Medicine, Centre for Pain Research and Galway Neuroscience Centre, National University of Ireland Galway, Galway, Ireland; 5 Hoba Therapeutics, Copenhagen, Denmark

**Keywords:** Anxiety, Chronic pain, Depression

## Abstract

Back-translating the clinical manifestations of human disease burden into animal models is increasingly recognized as an important facet of preclinical drug discovery. We hypothesized that inbred rat strains possessing stress hyper-reactive-, depressive- or anxiety-like phenotypes may possess more translational value than common outbred strains for modeling neuropathic pain. Rats (inbred: LEW, WKY, F344/ICO and F344/DU, outbred: Crl:SD) were exposed to Spared Nerve Injury (SNI) and evaluated routinely for 6 months on behaviours related to pain (von Frey stimulation and CatWalk-gait analysis), anxiety (elevated plus maze, EPM) and depression (sucrose preference test, SPT). Markers of stress reactivity together with spinal/brain opioid receptor expression were also measured. All strains variously developed mechanical allodynia after SNI with the exception of stress-hyporesponsive LEW rats, despite all strains displaying similar functional gait-deficits after injury. However, affective changes reflective of anxiety- and depressive-like behaviour were only observed for F344/DU in the EPM, and for Crl:SD in SPT. Although differences in stress reactivity and opioid receptor expression occurred, overall they were relatively unaffected by SNI. Thus, anxio-depressive behaviours did not develop in all strains after nerve injury, and correlated only modestly with degree of pain sensitivity or with genetic predisposition to stress and/or affective disturbances.

## Introduction

Standardisation of quantitative sensory testing in patients with peripheral neuropathic pain has revealed multiple clusters in which a principal sensory profile (e.g. sensory loss, mechanical hyperalgesia, thermal hyperalgesia) appears to link to distinct underlying disease mechanisms^1^. This could allow for defined populations of neuropathic pain patients to be recruited into clinical trials to help facilitate assessment of sensitivity to specific mechanism of action analgesics^2^. Accordingly, calls have been made for back-translational understanding of sensory testing in animal models of neuropathic pain, and also inclusion of non-evoked measurements, or assessment of parameters like sensory loss, or the emotional components of pain^3^.

Pain and emotion are tightly connected constructs. Stress, anxiety and depression are known to modulate pain perception both in the absence and presence of tissue injury^4–8^. Conversely, chronic pain has variously been reported to be intrinsically linked to an increased incidence of anxiety and depression in pain patients^9^. A growing body of evidence suggests that anxiety- and depressive-like behaviours may also occur in animal models of neuropathic pain^10^. However, the literature is far from conclusive and simple methodological discrepancies between studies, such as animal related differences, pain injury model or behavioural endpoints assessed, likely impact upon experimental outcome. Among other things, preclinical studies have shown that the age of the rodent^11^, choice of nerve injury model^12^, or even laterality/side of injury^13^ may affect development of comorbid emotional disturbances. And despite very similar methodology, some preclinical studies show time-dependent development of anxiety-like comorbidity following nerve-injury^14,15^, while others have failed to confirm this^16^, where the only apparent differences have been animal- and facility-related factors, like strain, gender and laboratory environment. A broad range of studies have now compared different inbred and outbred rat strains on parameters related to pain, anxiety and depression (examples:^17–24^), but surprisingly, to our knowledge, very few have explored the effects of strain/animal genetics on the development of emotional comorbidities following injury, despite assumptions that this may be a relevant factor to explain the variability in the field^25^.

When investigating behavioural correlates of pain and anxio-depressive comorbidity preclinically, either inbred mouse-strains, like C57BL/6, or outbred rat strains like Sprague–Dawley (SD) or Wistar are commonly used. Previous studies in our group have demonstrated clear strain-differences in sensory thresholds of naïve rats to nociceptive stimulation, and thereafter in their functional responsiveness to inflammatory- and neuropathic-injury^22,26^. Furthermore, we also reported that the μ-opioid receptor agonist morphine possessed distinct analgesic profiles across various inbred and outbred rat strains both in the absence and presence of tissue injury^26^. Taken together, these observations suggest that strains with genetic predisposition to stress hyper-reactivity, depressive- or anxiety-like phenotypes could possess a higher translational value when assessing emotional comorbid burden as experienced by human pain patients. However, this has never been tested empirically. Thus, the aim of the present study was to further characterize the sensory and emotional responses to peripheral nerve injury of these different inbred rat strains and compare with SD rats. Given the involvement of stress in pain, depression and anxiety^8,27–30^, we also assessed facets of stress reactivity of the included strains. Finally, a growing body of research has highlighted supraspinal changes in the opioid system as a potential link between pain and affect^8,31–35^. Therefore, we also decided to investigate plasticity within the opioid pain modulatory systems in the current study based on our knowledge of strain-dependent opioid-mediated analgesia^26^.

## Results

### Development of mechanical allodynia following nerve injury

**Figure 1:**
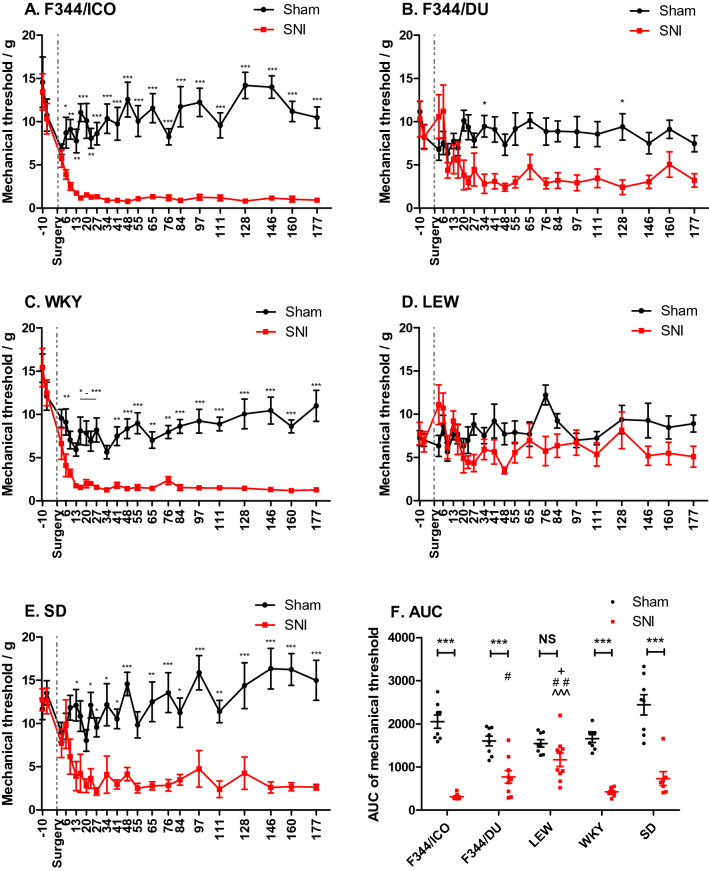
Development of mechanical allodynia in different rat strains following SNI. (**A**–**E**) 50% paw withdrawal thresholds to von Frey stimulation, calculated by the Dixon Up and Down method, were measured from 10 days prior to surgery until 177 days post-surgery. Dotted line marks day of surgery. Two-way Repeated Measures ANOVA with Bonferroni’s post-hoc test indicates difference between Sham vs. SNI for each time-point; *P < 0.05, **P < 0.01, ***P < 0.001. (**F**) Mechanical allodynia expressed as AUC in scatter plot format for each animal presented in (**A**–**E**). Two-way ANOVA demonstrated significant effects of strain (F [4,75] = 4.86, *P* = 0.0015), surgery (F [1,75] = 201.4, *P* < 0.0001) and a strain*surgery-interaction (F [4,75] = 9.895, *P* < 0.0001). Therefore Bonferroni post hoc testing was performed between individual strain- and surgical-groups, and not just on strain-level. Bonferroni’s post hoc test revealed differences between Sham vs SNI for each strain (NS = Not Significant = *P* > 0.05, *P < 0.05, **P < 0.01, ***P < 0.001), and for SNI-groups between strains (comparison with SD, ^+^*P* < 0.05; comparison with WKY, ^^^^^*P* < 0.001; comparison with F344/Ico, ^#^*P* < 0.05, ^##^*P* < 0.01). Data are presented as mean ± S.E.M.

The development of mechanical allodynia following SNI or sham-surgery for each strain is presented in Fig. 1A–E. Overall, repeated measures (RM) ANCOVA with second/last pre-surgical baseline as covariate, demonstrated overall significant effects of strain (F [4,74] = 5.697, *P* < 0.0001), surgery (F [1,74] = 166.3, *P* < 0.0001), and a strain*surgery interaction (F [4,74] = 8.639, *P* < 0.0001), indicating that the effect of surgery was not similar for all strains. We therefore performed two-way RM ANOVA for each individual strain, and found the effect of surgery to be significant for all strains, except for the Lewis strain (F_F344/ICO_[1,330] = 94.24, *P* < 0.0001. F_F344/DU_[1,330] = 11.57, *P* = 0.0039. F_WKY_[1,352] = 134.7, *P* < 0.0001. F_SD_[1,286] = 25.98, *P* = 0.0002). For the majority of the strains, surgery was the main parameter explaining the variation, most prominently in F344/ICO, and the least in LEW (F344/ICO: 50.76% > SD: 35.59% > WKY: 32.51% > F344/DU: 13.62% > LEW: 3.26%,).

The development of mechanical allodynia during the entire test period was also studied by calculating Area Under the Curve (AUC) values for each animal (Fig. 1F). Two-way ANOVA demonstrated overall significant effects of surgery (F [1,75] = 201.4, *P* < 0.0001), strain (F [4,75] = 4.860, *P* = 0.0015) and their interaction (F [4,75] = 9.895, *P* < 0.0001). Bonferroni’s posthoc test revealed that AUC for SNI-groups were significantly lower than sham for all strains (*P* < 0.001), with the only exception being LEW where sham and SNI groups were not statistically different (Fig. 1F). In addition, when comparing sham-groups, the F344/ICO strain was significantly higher than F344/DU and LEW strains (*P* < 0.05), whilst F344/DU, LEW and WKY were all significantly lower than the SD strain (*P* < 0.001).

SNI-surgery had no impact on 50% mechanical response threshold in the contra-lateral paw at the end of the study, but there were significant effects of strain, (F [4,75] = 9.360, *P* < 0.0001, two-way ANOVA) (Supplementary Fig. S1A). Comparing ipsi- and contra-lateral thresholds at the end of the study, showed a statistically significant effect of surgery (F [1,75] = 46.68, *P* < 0.0001), but also a strain*surgery interaction (F [4,75] = 2.669, *P* = 0.0386, two-way ANOVA), again indicating that surgery had more effect for some strains than others (Supplementary Fig. S1B). Bonferroni’s post-hoc test confirmed this, as there were no significant differences between sham vs SNI for LEW and F344/DU, contrary to the other strains (Supplementary Fig. S1B).

### Functional gait-changes following nerve injury

**Figure 2:**
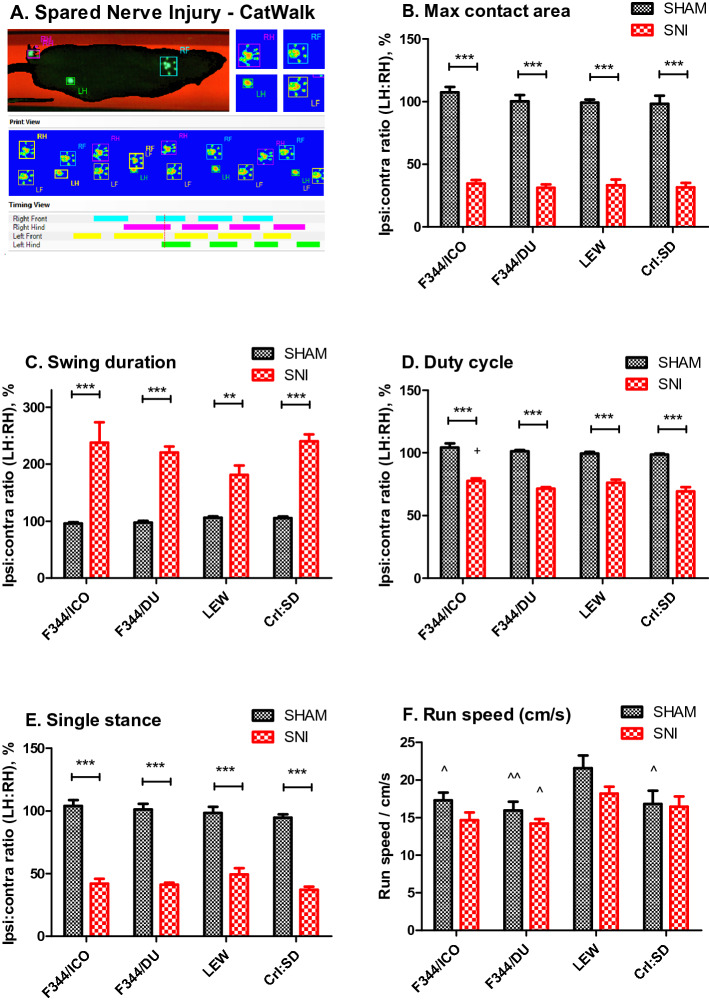
Functional gait impairment develops in all rat strains after SNI. (**A**) Spared Nerve Injury—CatWalk: Image of a LEW SNI-rat walking on the CatWalk. In the ‘timing view’ in the bottom, the green lines indicate the timing of the injured hind-limb being in contact with the glass plate, while the pink lines indicate contact with the uninjured hind-limb. Comparing these demonstrates increased duration of contact with the surface for the uninjured limb, compared with the injured, while the print representations above (RH, RF, LH, LF) shows the print-area in contact with the plate. Notice that the injured hind paw (LH: Left Hind) has a much smaller print-area, primarily with the heel of the paw. (**B**) Maximum contact area (cm^2^); the maximum surface area of a paw that comes into contact with the glass plate. Presented as a ratio between injured and uninjured hind-limbs. (**C**) Swing (s): swing or swing phase is the duration in seconds of when a paw is not in contact with the glass plate. Presented as a ratio between injured and uninjured hind-limbs (**D**) Duty cycle (%): (stand/(stand + swing)*100%. Presented as a ratio between injured and uninjured hind-limbs. Bonferroni post-test indicates significant difference between ICO-SNI vs SD-SNI (^+^*P* < 0.05). (**E**) Single stance (s): the duration of ground contact for a single hind paw, where the contralateral paw is not on the plate. Presented as a ratio between injured and un-injured hind limbs. (**F**) Run speed, cm/s. General differences in speed on the CatWalk. Data are presented as mean ± S.E.M. Difference between Sham vs. SNI within the same strain; **P < 0.01, ***P < 0.001, and for comparison with surgery-specific groups between strains; ^^^*P* < 0.05, ^^^^*P* < 0.01 for comparison with LEW, determined by two-way ANOVA and Bonferroni’s post test.

To detect if functional gait abnormalities occurred following SNI, all strains were assessed on the CatWalk as illustrated in Fig. 2A. For purposes of simplicity, we chose to focus only on a selected number of parameters relevant to gait and coordination in accordance with previous studies that have used this method to assess functional outcome after nerve injury in rats^36–39^. In general, WKY rats were unwilling to walk across the platform despite multiple attempts to facilitate the process, and we only obtained data for three animals per group (SNI vs sham) for this strain. Therefore, the WKY strain were excluded from the final data analysis albeit the few animals tested showed the same overall trends for the measured parameters as the other strains presented in Fig. 2B–F.

Overall, we observed a significant effect of surgery for the hind paw contact area (F [1,59] = 526.1, *P* < 0.0001, two-way ANOVA, strain*surgery), swing duration (F [1,59] = 105.3, *P* < 0.0001), single stance ratio (F [1,59] = 394.8, *P* < 0.0001) and duty cycle (F [1,59] = 427.0, *P* < 0.0001), which compares the stand phase with the entire step cycle ((stand/(stand + swing))*100%), (Fig. 2B–E). No strain-differences were detected for contact area, swing duration or single stance, but for the duty cycle, there was a significant effect of strain (F [3,59] = 3.558, *P* = 0.0196, two-way ANOVA, strain*surgery). Exploring the overall run speed of the voluntary movement across the CatWalk for the different strains and surgical groups, as shown in Fig. 2F, revealed significant effects of both strain (F [3,59] = 6.236, *P* = 0.0009) and surgery (F [1,59] = 5.551, *P* = 0.0218). However, post hoc analysis failed to show any significant difference on this latter parameter between SNI and sham-controls for the individual strains, indicating that there did not appear to be an overwhelming deficit in the general activity or ability to move of SNI rats.

### Development of anxiety-like behaviour following nerve injury

**Figure 3:**
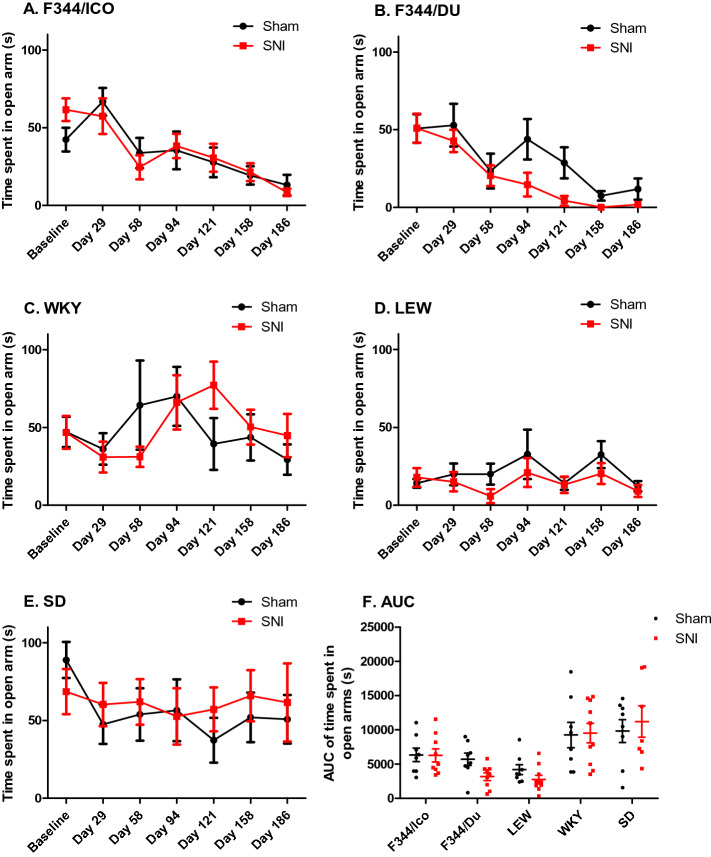
Anxiety behavior in different rat strains is minimally affected by SNI. (**A**–**E**) show the time spent in the open arms of the elevated plus maze for each rat strain at baseline and up to Day 186 post SNI. Two-way Repeated Measures ANCOVA, covariate = baseline, showed significant effect of surgery for F344/DU. Bonferroni’s post test, showed no significant difference between Sham vs. SNI for F344/DU at individual timepoints. (**F**) AUC of time spent in open arms. Two-way ANOVA showed significant effect of strain (F [4,75] = 11.621, *P* < 0.0001), and Bonferroni’s post hoc test showed that SD spent significantly more time in the open arms than LEW, F344/Du and F344/ICO (*P* < 0.001), and WKY spent more time there than LEW and F344/Du (*P* < 0.001), as detected pairwise combined for the two surgical groups for each strain, given that there were no overall effect of surgery on the AUC. Data are presented as Mean ± S.E.M.

To assess the possible development of anxiety-like behaviour after neuropathic injury, rats were tested on an Elevated Plus Maze (EPM) at baseline, and once a month after surgery (Fig. 3 and Supplementary Fig. S2). RM ANCOVA (surgery*strain, covariate = baseline) for each individual area of the maze, showed significant effects of strain for time spent in the open arms (F [4,74] = 7.482, *P* < 0.001, Fig. 3), closed arms (F [4,74] = 18.346, *P* < 0.001, Supplementary Fig. S2) and centre zone (F [4,74] = 28.243, *P* < 0.001, data not shown). A trend was observed for an interaction between surgery and strain for time spent in both the open (F [4,74] = 2.176, *P* = 0.080) and closed arms (F [4,74] = 2.414, *P* = 0.056), indicating that surgery may have induced anxiety-like behavior in some strains. Analysis of the effects of sham vs SNI for individual strains showed that only F344/DU rats exhibited significant effects of surgery on the amount of time spent in both closed (F [1,14] = 6.084, *P* = 0.027, RM ANCOVA, covariate = baseline) and open arms (F [1,14] = 9.999, *P* = 0.007), indicating increased anxiety-like behaviour as a result of the peripheral nerve injury. Overall, regardless of surgical groups, both LEW and F344 strains spent less time exploring the open arms across the time course of the study, either due to repeated testing, aging, or increased anxiety-levels across strains and surgical groups (F_F344/ICO_[6,90] = 8.684, *P* < 0.0001. F_F344/DU_[6,90] = 25.53, *P* < 0.0001. F_LEW_[6,96] = 6.169, *P* < 0.0001, two-way (surgery*time) RM ANOVA).

Finally, collapsing the raw time course data into Area Under the Curve (AUC) values (Fig. 3F and Supplementary Fig. 2F) confirmed the presence of significant strain-effects on time spent in the different parts of the maze (F_open_[4,75] = 11.63, *P* < 0.0001. F_closed_[4,75] = 42.78, *P* < 0.0001, two-way ANOVA (strain*surgery)).

### Development of depressive-like behaviour following nerve injury

**Figure 4:**
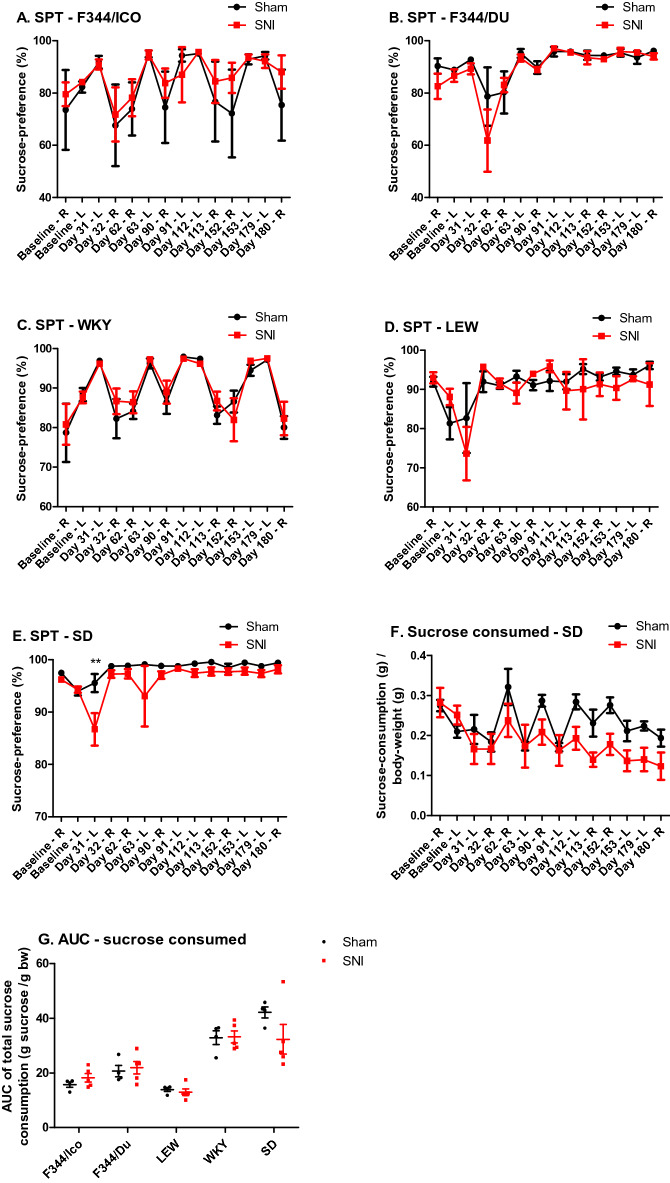
Sucrose preference in different rat strains is minimally affected by SNI. (**A**–**E**) Sucrose consumption expressed as a percentage of total fluid consumption as an index of anhedonia. Notice that this parameter is recorded on cage level, not subject level, and therefore N = 5 for SNI-groups, N = 4 for Sham groups. Sucrose consumption was measured on two consecutive test-days, where the sucrose bottle was placed in either the “L” = left or “R” = right side of the cage. Only SD showed significant effects of surgery. Bonferroni’s post comparison test showed significant differences between Sham and SNI-injured animals only on Day 31 post injury, **: *P* < 0.01. (**F**) Sucrose consumption expressed compared to body weight for SD, confirmed the decreasing sucrose consumption for SNI compared with Sham. (**G**) AUC of total amount of sucrose consumption related to body weight. Two-way ANOVA showed significant effect of strain (F [4,45] = 30.891, *P* < 0.0001), and Bonferroni’s post hoc showed that both the SD- and WKY-strain consumed significantly more than LEW, F344/Ico and F344/Du (*P* < 0.001), and that F344/Du consumed more than LEW (*P* < 0.05), as detected pairwise combined for the two surgical groups for each strain. Data are presented as mean ± S.E.M.

To assess the possible presence of depressive-like behaviour in SNI rats we used the Sucrose Preference Test (SPT) as a measure of anhedonia (Fig. 4A–E). For some strains, there were clear variations in sucrose preference between tests days, which depended upon which side of the cage the sucrose bottle was presented. Therefore, we analyzed the data using RM ANCOVA, (side*strain*surgery, covariate = baseline), in order to factor in both side- and side-specific baselines, as the measure was performed for 24 h in both sides for each timepoint. The analysis showed significant effect of ‘side’; (F [1,69] = 20.055, *P* < 0.001), and an interaction between side*strain (F [4,69] = 2.526, *P* = 0.048), indicating that the side-factor had stronger impact for some strains than others, despite all strains having both bottles permanently present in the home-cage throughout the 6 month study. However, there was no significant effect of surgery when analyzing the full dataset in this way.

Thereafter, when analyzing each strain individually (RM ANCOVA, side*surgery, covariate = baseline), the two F344 strains and WKY rats all showed a significant effect of side (F_F344/ICO_[1,13] = 6.043, *P* = 0.029, F_F344/DU_[1,13] = 9.051, *P* = 0.010, F_WKY_[1,13] = 33.676, *P* < 0.001), (Fig. 4A–C) in contrast to the LEW and SD strains (Fig. 4D,E). However, a significant effect of surgery on sucrose preference percentage was only observed in SD rats (F_SD_[1,13] = 13.281, *P* = 0.003). This finding was further confirmed when presenting the SPT data as the total amount of sucrose consumed (sucrose (g) / body weight (g)) (F_SD_[1,13] = 44.792, *P* < 0.001, Fig. 4F), in contrast to the other strains which are presented as AUC in Fig. 4G and in full in Supplementary Fig. S3. Notably, when presented as the amount consumed rather than the percentage compared with water consumption, the effect of cage side disappeared for all strains (RM ANCOVA, strain*surgery*side, covariate = side-specific baseline). Although significant effects of surgery (F [1,69] = 10.540, *P* = 0.002) and strain (F [4,69] = 16.965, *P* < 0.001) emerged, there was also a prominent strain*surgery-interaction (F [4,69] = 9.621, *P* < 0.001), indicating that the surgery-effect was not similar across strains. Finally, Bonferroni pairwise comparisons showed that SD rats consumed significantly more sucrose-water related to body weight than F344/DU (*P* = 0.025), F344/ICO (*P* = 0.049) and LEW (*P* = 0.009), and that WKY rats consumed more than all the other inbred strains (*P* < 0.001), which was also seen when presenting the data as AUC for the full time-duration (Fig. 4G). The overall development of body weight during the study, was also strain-dependent, and is presented in Supplementary Fig. S4. Since the SPT was performed on cage-level, each measurement represents the two animals that were housed together (and receiving the same surgery), and ‘sucrose consumed’ was calculated based on the total body weight in the cage compared to the amount consumed for the cage. Unfortunately, three test-subjects from the SD-SNI-group were lost, leading to 3 subjects unintentionally being single-housed (3 cages) and 4 pair-housed (2 cages) from this group. As social isolation may impact the development of anhedonia^40^, the two sub-groups of SD-SNI animals (single- vs. pair-housed) was also compared (Supplementary Fig. S5), but given the very low number per group, statistical comparisons could not confirm that housing was contributing to the development of anhedonia.

### Fecal corticosterone and immunoreactive corticosterone metabolites and organ weights

**Figure 5:**
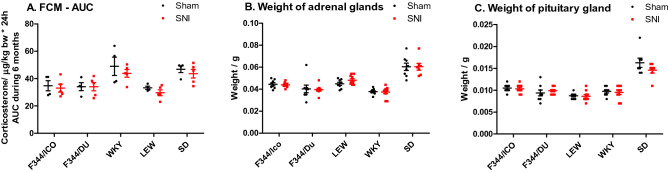
HPA-axis activity in different rat strains after SNI. (**A**) Excretion of Fecal Corticosterone Metabolites (FCM)—AUC. The full time-course is presented in Supplementary Fig. S4. FCM was measured at baseline, 1, 2, 4 and 6 months post-surgery. AUC was calculated for each individual/cage. Note that this parameter is recorded on cage-level, not subject-level, and N = 5 for SNI-groups, N = 4 for Sham groups. Two-way ANOVA showed significant effects of strain with Bonferroni’s post testing showed that both the SD—(*P* < 0.05–0.001) and WKY-strain (*P* < 0.01–0.001) had significantly higher FCM-levels than F344/Ico, F344/Du and LEW, as detected pairwise combined for the two surgical groups for each strain. (**B**) Weight of adrenal glands. (**C**) Weights of pituitary gland for each animal. Notice that the adrenal and pituitary weights presented in panels (**B**,**C**) are potentially affected by the size of the animal, explaining why the statistical analysis was performed with body weight as a covariate. The analysis showed significant effect of strain (two-way ANCOVA, strain*surgery, covariate = body weight) for both organs, and Bonferroni’s post hoc test detected differences between strains. For adrenal weights; SD had significantly higher adrenal weight than F344/Du (*P* < 0.05) and WKY (*P* < 0.001), and WKY also having smaller adrenal weights than F344/Ico (*P* < 0.01) and LEW (*P* < 0.001), when body weight was included as a covariate. For pituitary, SD had significantly higher weight/size than WKY (*P* < 0.05) and LEW (*P* < 0.001), and F344/Ico had higher pituitary weights than LEW (*P* < 0.001). Given the lack of statistical effect of surgery or strain*surgery interaction, the post hoc differences are presented overall between strains, and not divided for the surgical groups. Data are presented as scatter plot and lines indicating mean ± S.E.M.

Fecal corticosterone and immunoreactive corticosterone metabolites (FCM) were measured in order to detect putative effects of peripheral nerve injury on hypothalamo-pituitary-adrenal (HPA)-axis function. FCM was measured at baseline, 1, 2, 4 and 6 months post-surgery, and presented as collected AUC for the study duration for each cage in Fig. 5A, and in full in the Supplementary Fig. S6. RM ANCOVA (strain*surgery, covariate = baseline) detected a clear effect of strain (F [4,34] = 5.217, *P* = 0.002), but not surgery. It also showed a significant difference between LEW and two of the strains; WKY (*P* = 0.022) and SD (*P* = 0.005, Bonferroni’s post-hoc test). Comparing AUCs for the entire study period again confirmed the significant effect of strain (F [4,35] = 8.998, *P* < 0.0001, two-way ANOVA), but not surgery (Fig. 5A). It also showed that WKY and SD excreted significantly more fecal corticosterone than the other strains.

As an additional gross measure of HPA-axis activity, we measured the weight of the adrenal and pituitary glands at the end of the study (Fig. 5B,C respectively). For both organs, data are shown as the actual organ weights, while the statistical analysis was made with the body weight as a covariate, to incorporate the potential effect of the animal’s size on the organ weight. Statistical analysis showed no effects of surgery on organ weights, but there were clear effects of strain (F_adrenal_[4,74] = 9.078, *P* < 0.001, F_pituitary_[4,73] = 10.514, *P* < 0.001, two-way ANCOVA, strain*surgery, covariate = bodyweight). Body weight was found to be a significant factor only for the pituitary (F_pituitary_[1,73] = 6.975, *P* = 0.011). Notably, WKY rats had a significantly lower adrenal weight (when body weight was a covariate), compared to the LEW, SD and F344/ICO strains (*P* < 0.001–0.008) (Fig. 5B). However, the pituitary glands were significantly larger for F344/ICO and SD strains compared with LEW rats (*P* < 0.001), and for SD compared with WKY rats (*P* = 0.025, two-way ANCOVA and Bonferroni’s post-hoc test).

### Western blotting for opioid receptors

**Figure 6:**
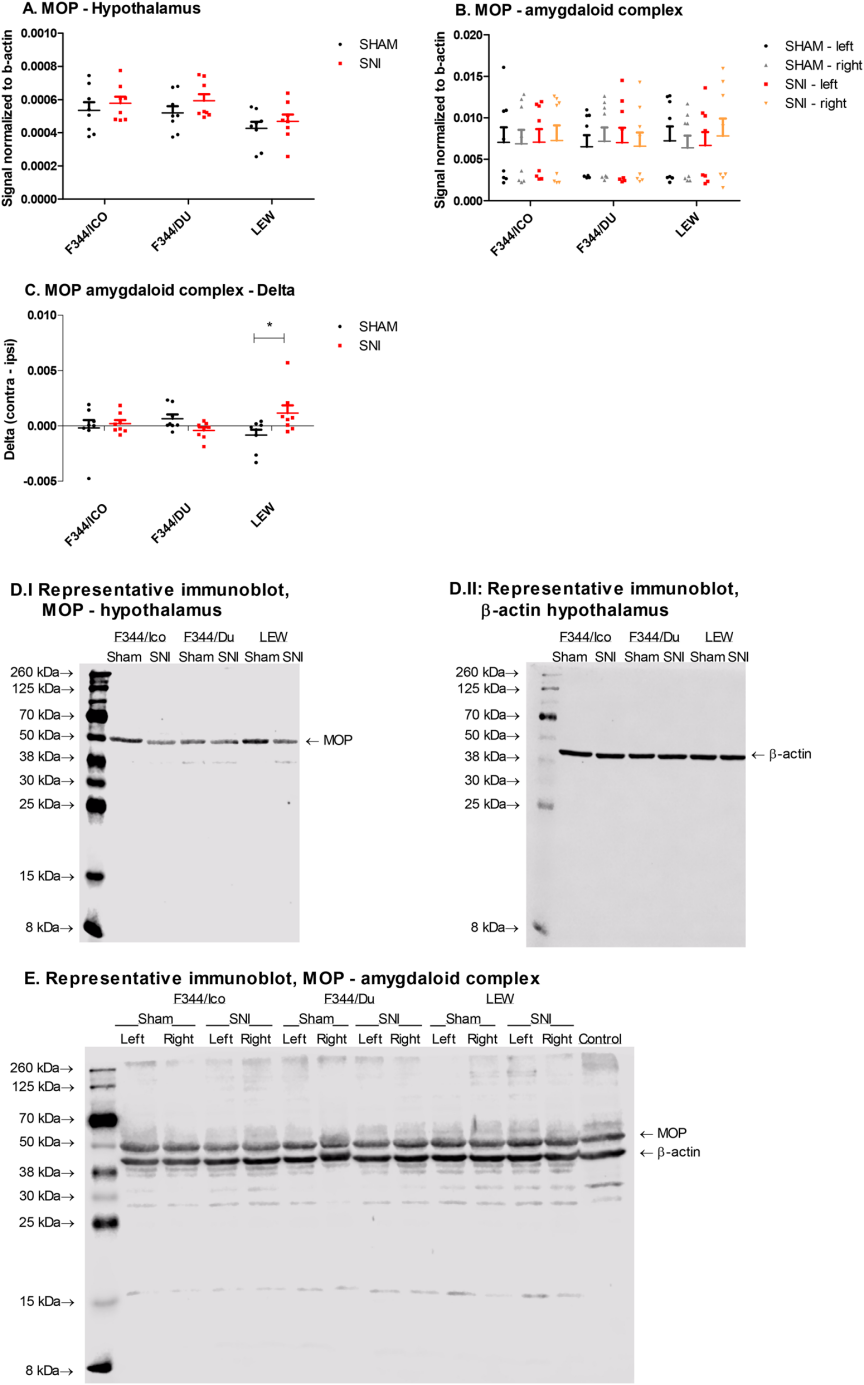
MOP expression in brain and spinal cord of selected rat strains following SNI. (**A**) Expression of MOP in the hypothalamus. Two-way ANOVA showed significant effects of strain, but no differences in Bonferroni’s post-hoc test. (**B**) Expression of MOP in left and right amygdaloid complex. (**C**) Expression of MOP, expressed as the difference between right and left amygdaloid complex. Two-way ANOVA showed significant strain*surgery-interaction, and a significant difference between SNI and Sham for LEW (*P* < 0.05, Bonferroni’s post-hoc test). (**D**,**E**) Representative full length immunoblots of hypothalamus (MOP; **D.I,** β-actin; **D.II**) and amygdaloid complex (**E**), presenting the MOP-bands at ~ 53 kDa, and β-actin at ~ 42 kDa. Data are presented as mean ± S.E.M. of the signal normalized to beta-actin in the sample. N = 8.

µ opioid receptor (MOP) expression was measured within the hypothalamus, rostral ventromedial medulla (RVM), periaqueductal grey (PAG), dorsal part of the lumbar enlargement of the spinal cord (ipsi- and contra-lateral), left and right amygdaloid complex and prefrontal cortex (PFC) (Fig. 6 and Supplementary Fig. S7). Within the amygdaloid complex, statistical analysis revealed a strain*surgery-interaction (F [1,41] = 4.172, *P* = 0.022), two-way ANCOVA, strain*surgery, covariate = right amygdala) (Fig. 6B). Notably, the strain-dependent increase in MOP expression after SNI was even clearer, when the data were presented as the difference (delta) between left and right amygdala, (Interaction: F [2,42] = 4.437, *P* = 0.0179, two-way ANOVA, strain*surgery), and confirmed by Bonferroni’s post-hoc test (LEW SNI vs SHAM, *P* < 0.05) (Fig. 6C). Within the hypothalamus, we also detected a significant effect of strain (F [2,42] = 4.565, *P* = 0.0161, two-way ANOVA, strain*surgery) on MOP expression with a 17.2–20.2% reduction observed in the LEW strain compared with the two F344-substrains for both sham and SNI groups (calculation: ((F344-LEW)/F344)*100) (Fig. 6A). Otherwise, there were no significant effects of strain or surgery on the level of MOP within PAG, RVM, PFC or spinal cords, and so these remaining results are presented in the supplementary Figure S7.

**Figure 7:**
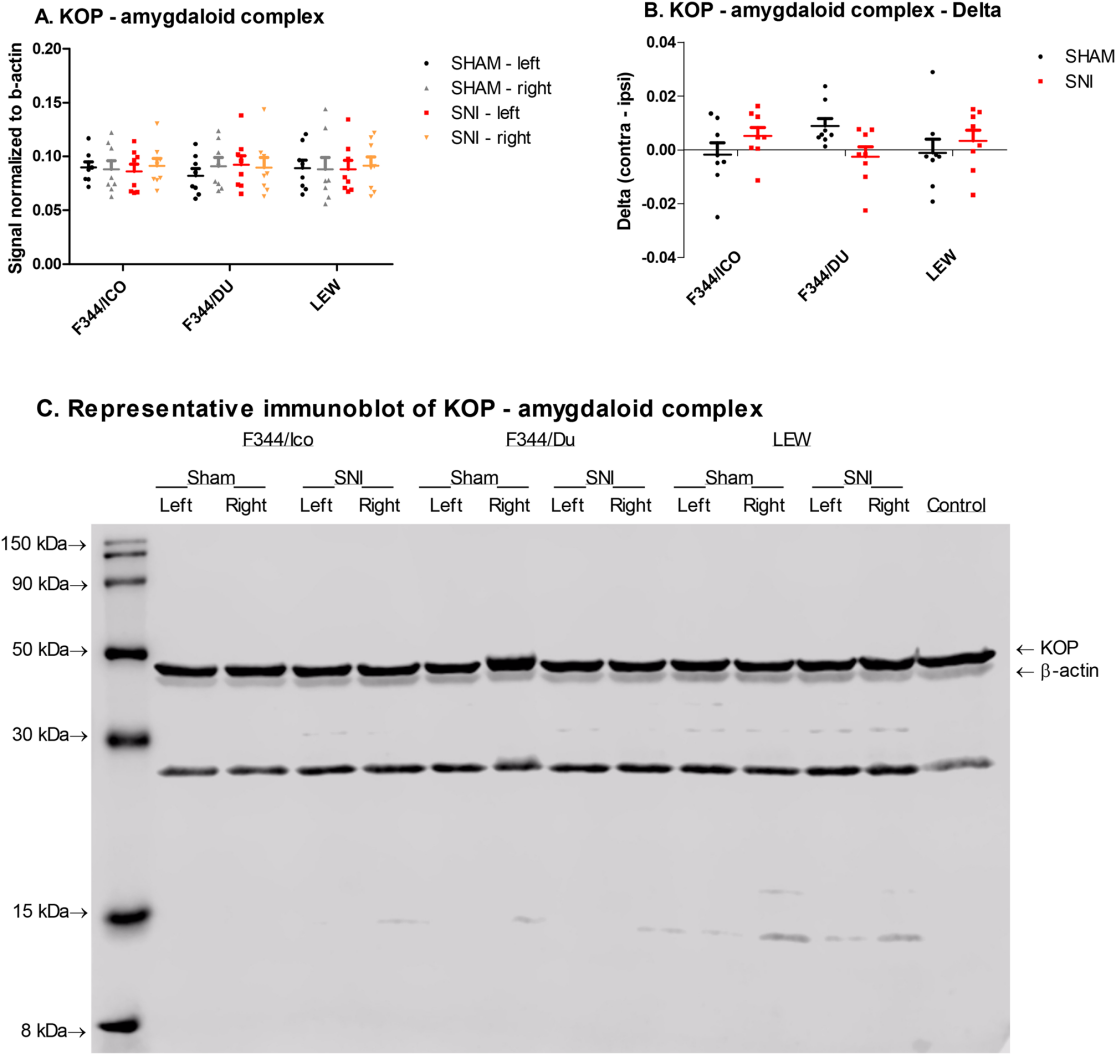
KOP expression in brain and spinal cord of selected rat strains following SNI. (**A**) KOP in the amygdaloid complex. Two-way ANCOVA, strain*surgery, covariate right amygdaloid complex, showed significant strain*surgery-interaction. (**B**) KOP in the amygdaloid complex, expressed as the difference between right and left amygdaloid complex. Two-way ANOVA showed an almost significant strain*surgery-interaction. (**C**) Representative full length immunoblots of the amygdaloid complex, presenting the KOP-bands at ~ 43 kDa, and β-actin at ~ 42 kDa. The unspecified band at ~ 25 kDa was apparent after staining with the KOP-antibody, and likely represents degraded forms of the native KOP. Data are presented as mean ± S.E.M. of the signal normalized to β-actin in the sample. N = 8.

κ opioid receptor (KOP) expression was measured within the amygdaloid complex (left and right) (Fig. 7), PFC and the dorsal part of the lumbar enlargement of the spinal cord (ipsi and contra) (Supplementary Fig. S8). Similar to MOP, we detected a strain*surgery-interaction (F [1,41] = 4.012, *P* = 0.026, two-way ANCOVA, strain*surgery, covariate = right amygdala) (Fig. 7A), which became visually clearer when expressed as delta-values (Interaction: F [2,42] = 3.201, *P* = 0.05, two-way ANOVA, strain*surgery) (Fig. 7B). No statistical differences were detected in the PFC and spinal cords, and they are therefore only presented in the supplementary file (Supplementary Fig. S8).

## Discussion

Great efforts are being made to optimise translational read-outs from bench to bedside across the pain landscape^41^. Notably, concerns have been raised that animal studies primarily rely on nociceptive withdrawal reflex based assays instead of endpoints incorporating facets of spontaneous pain or sensory loss, functional impairment, and emotional aspects of the pain condition^3,42,43^. Using a selection of inbred rat strains that possess a genetic predisposition to stress hyper-reactivity, depressive- or anxiogenic-like phenotypes, we have explored their long-term propensity towards expressing sensory and emotional disturbances following peripheral nerve injury. Extending on our previous study^22^, the current work also included, (1) a more prolonged time-course, (2) assessment of pain- and affective-endpoints in the same subject, (3) assessment of functional gait-deficits, (4) sham-control groups, (5) additional F344-substrains, to elucidate the effects of not only inbred strains, but substrains thereof, (6) fecal corticosterone measurements and organ weights as proxy markers for HPA activity, and finally (7) assessment of plasticity within the opioid system following long-term nerve injury.

### Allodynia and gait disturbances after SNI

Four of the five rat strains tested in the current study, variously developed mechanical allodynia as a consequence of SNI (F344/ICO = WKY = SD > F344/DU), in agreement with reports from other laboratories^44–46^. Although the clear lack of mechanical hypersensitivity after neuropathic injury in the stress-hyporesponsive LEW strain is consistent with a recently published study from our group^22^ and others^46,47^, gain of sensory function has also been reported^19,48,49^. Interestingly, the two F344 sub-strains, exhibited markedly different responses to hind paw mechanical stimulation throughout the study and highlight that sub-strain differences may be one of the explanations for discrepant findings between research laboratories. Notably, even for inbred rat strains, accumulated mutations and genetic drift are expected to produce variability between subpopulations^50–52^. Similarly, we have previously demonstrated clear differences in development of neuropathic allodynia and pharmacologic sensitivity between outbred SD sub-strains^53^.

Although the underlying genotype of the strains tested herein would appear to provide the simplest explanation for the associated neuropathic phenotype, it is possible that deviances in the surgical protocol due to strain-related differences in nerve anatomy and/or innervation might have been a possible contributory factor^54^. However, we think this is unlikely since all strains displayed similar SNI-specific postural changes with pronation of the affected paw^55,56^. Moreover, increased sensitivity to evoked stimuli is merely one sign amongst a myriad of sensory changes experienced by neuropathic patients^57^. A more predominant symptom for neuropathic pain patients is spontaneous pain, which although more difficult to measure in animals^43,58^, might be expected to improve the translational utility of pre-clinical pain data^59^. Accordingly, dynamic gait-alterations occurring as a consequence of injury in rodents have been suggested to represent a surrogate marker of spontaneous pain^60,61^. In our experiments, CatWalk analysis revealed that SNI rats from all strains displayed functional gait impairment irrespective of the presence of evoked mechanical allodynia. All strains moved with greater awareness for the affected limb and tried to minimize contact and pressure of the injured paw with the ground, but without profound impact on their willingness or speed of movement. Importantly, our data are generally consistent with similar studies exploring dynamic gait-changes produced by peripheral nerve injury in rodents^36,37^. Whether these changes simply represent secondary biomechanical effects of nerve injury, muscle atrophy or reflect facets of spontaneous pain or sensory loss, would be interesting to test in future pharmacology studies using standard of care analgesics.

### Emotional disturbances after SNI

Although many preclinical studies have shown that neuropathic pain correlates positively with anxio-depressive behaviour^13–15,31,62–69^, this outcome has been far from consistent^16,70–75^. Time appears to be an important factor, as anxiety-behaviour rarely presents within the first couple of weeks after injury^70,71,73^, requiring 4 weeks or more to manifest^65,67,76,77^. Depressive-like behaviour may require even more time to evolve^66^. Whilst some studies suggest that up to 4 months may be required before robust anxiety-behaviour is evident after injury^14,15^, other studies using similar methodology have not replicated these observations^16^, perhaps as a consequence of animal-related factors such as strain or gender^25^.

To our knowledge, very few studies have explored the effect of strain on the development of emotional comorbidities after nerve injury^44^. The Wistar Kyoto (WKY) rat was first developed as the counterpart to the spontaneous hypertensive rat (SHR) strain, and has been reported to possess both ‘depressive-’^78^, and ‘anxiety-like’ phenotypes^79^, as well as distinct changes in hypothalamo-pituitary-adrenal (HPA) axis function^80,81^, where they have been described as being stress hyperresponsive^82^. The inbred Lewis (LEW) and Fischer (F344) strains have typically been used to investigate aspects relating to HPA-function based on their respective hypo- and hyper-reactive responses to stress^83–85^. Moreover, similarly to WKY rats, LEW rats have been suggested as a high-anxiety counterpart to the SHR strain when modeling high and low indices of basal anxiety^21,86,87^. Despite detecting very prominent differences in evoked pain-like behaviour between strains, we did not see any correlation between magnitude of neuropathic pain-like behaviour and affective changes indicative of the presence of anxiety- or depressive-like behaviour, confirming other recent findings^33,88^. Notably, the only strain showing anxiety-like behaviour in the EPM following nerve injury was the moderately pain-sensitive F344/DU. Similarly, only SD rats showed indications of anhedonia after nerve injury, and most prominently when assessed as the amount of sucrose consumed, similar to recently reported^33^. The sucrose preference test is a commonly used non-invasive test of anhedonia / depressive-like behaviour used in rodents^89^. Contrary to traditional tests of depressive-like behaviour, like the Forced Swim test, it is not based on locomotor action/ability, may be recorded in the home-cage environment, and has previously been sensitive enough to detect a shift in preference^90–93^ or amount consumed^33^ in chronically nerve-injured rodents. The sensitivity to the sucrose preference test has though been found to vary with strain^20,94^. This was clearly the case in the current study, where the three stress-hyperresponsive strains (WKY, F344/ICO & F344/DU) all showed very prominent effects of ‘side’ of the cage, that the sucrose bottle was presented in. Although it was attempted to control for this effect by using the side-specific baseline as covariate in the analysis, and converting the data to ‘amount consumed’, this assay limitation may well have masked any possible modifying effects of nerve-injury on the putative expression of depressive-like behaviour. The aim was to secure minimal stress by maintaining the animals in their undisturbed home-environment with their cohoused partner during the test, but as testing on cage/pair-level lead to smaller group-sizes, and also the possibility, that a side-preferring subject would affect the outcome of a pair, it could be indicated to incorporate the following adjustments for future studies; i) short-term water-deprivation, ii) temporary single-housing for the actual test, but next to cage-partner and in home environment, iii) testing during the night-phase, iv) testing on two consecutive nights with the sucrose-bottle in each side. An additional limitation of the present study was that only one test was used for anxiety-related behavior (EPM) and depression-related behaviour/anhedonia (SPT), and at discrete time points post-SNI. We cannot exclude the possibility that additional alterations in anxio-depressive behaviour, and correlations with pain sensitivity, might have been revealed with additional assays and/or different time points post-injury. Moreover, our results and conclusions are limited to SNI, and cannot necessarily be extrapolated to other models of neuropathic pain.

Physical activity and a socially enriched environment have been found to reduce pain-like behaviour and normalize brain function in rodents^95^. Thus, it has commonly been suggested that environmental enrichment should be minimized and rodents single-housed when used as models for depressive- or anxiety-like behaviour, to ‘enhance’ the behavioural phenotypes^40,94^. Nevertheless, we purposefully decided not to follow this strategy, as we wanted the neuropathic pain condition to be the only factor affecting the animals. During the current study, three test-subjects from the SD-SNI-group were lost, which left 3 subjects being unintentionally single-housed and 4 subjects pair-housed from this group. However, since statistical analysis could not confirm if single-housing was a significant factor, likely related to low group size (Supplementary Fig. S4), both single and pair-housed animals were included equally. It is therefore interesting that anhedonic behaviour in the SPT is only detected in this strain, especially as SD have previously been reported to be more sensitive to single-housing than F344 and LEW in a Chronic Mild Stress model^96^, and it remains uncertain to which extent the single-housing is driving the anhedonia-like behaviour in the SD SNI-group.

### Pain modulatory systems after SNI

A wide range of molecular mechanisms have been explored to help explain the emotional comorbidities related to chronic pain (for review, see^35,97^). Amongst these is the HPA axis, which plays a central role in the homeostatic control of the stress response and contributes to the clinical aetiology of anxiety and depression^27^. However, chronic neuropathic pain injury alone does not appear to robustly influence HPA axis function in rodents^98,99^, and so we were not surprised at the lack of effect of neuropathic injury on the biomarkers and organ weights measured. Again, the general lack of a positive correlation of neuropathic pain with comorbid mood disturbance in our behavioural experiments aligns with the apparent lack of change in markers of HPA axis activation for the strains included here, and a previous study detecting anxio-depressive comorbidities in neuropathic pain, also found that the HPA-axis was unchanged^66^. Moreover, despite the established use of LEW and F344 rats as models of stress hypo- and hyper-responsivity, they possess similar corticosterone and ACTH serum levels under baseline non-stressful conditions^85,100^, consistent with the observed FCM levels in our experiment. Importantly, WKY rats presented with higher FCM levels than the other inbred strains, and greater adrenal weights compared with LEW, SD and F344/ICO strains confirming that lack of assay sensitivity was unlikely to be responsible for the lack of effect of neuropathic injury on HPA axis function.

The endogenous opioid system is involved in the physiology of stress, nociception, anxiety and depression^8,31,101^. The PAG and RVM are important components of descending pain pathways, and receive inputs from sites including the amygdala and hypothalamus^102^. During neuropathic pain, tonic descending inhibitory transmission is replaced by increased drive from descending facilitatory pathways which further enhance spinal excitability^103^, and differences in these pathways, and especially within the amygdala, PAG and RVM, have been found to explain differences in neuropathic and inflammatory pain sensitivity between rat strains^23,24,104–106^. Opioid-mediated signaling mechanisms in these pathways contribute to the manifestation of neuropathic hypersensitivity after injury^107^, and are related to the affective disturbances arising in relation to pain^10,31^. However, changes in opioid receptor expression within the spinal dorsal horn could not be detected in the current study, suggesting that behavioural differences between strains and surgical groups originate supraspinally, or are unrelated to changes in this endogenous system. Supraspinal opioid receptor expression levels have though previously been found to be reduced following nerve injury and to be directly related with emotional comorbidities^33,34^. Similarly, sustained pain conditions induce a reduction in opioid receptor in the amygdala^108^, and lateralized opioid-signaling can develop, generally with indications of a pronociceptive role of the right amygdala^32^. Interestingly, for both opioid-receptors (KOP and MOP) measured in the amygdaloid complex, there were significant strain*surgery-interactions, when factoring in the difference between left and right amygdala expression levels, most prominently with a shift in MOP-expression to the right amygdaloid complex for nerve-injured LEW, while the trend was opposite for nerve-injured F344/DU rats. Increased expression in the right amygdala may be connected to previous findings of enhanced evoked activity in the right central amygdala following nerve-injury, unrelated to side of peripheral injury^109^, while the shift to left amygdaloid complex for F344/DU may relate to reports that the left amygdala undergoes changes associated to anxiety-disorders^110^. A significant effect of strain on MOP-expression was detected only in the hypothalamus, where LEW generally expressed lower levels compared with the F344-substrains. Previous findings have indicated less binding and functionality of MOP in various brain-areas, including PAG and amygdala, for LEW than F344^111^, but surprisingly, the strain-difference in MOP-expression was only present in hypothalamus in the current study.

In summary, the current study showed clear differences in development of pain-, and to a lesser degree depressive- and anxiety-like behaviours in response to peripheral nerve injury (SNI) in different inbred rat strains, despite similar dynamic gait-changes. Thus, different rat (sub-) strains appear to develop distinct symptomatic and sensory phenotypes after neuropathic injury, which may have important implications in light of the recent ‘call for back-translation of sensory profiling in animal models of neuropathic pain’^3^. Crucially, the strain-dependent development of anxio-depressive comorbidities related to chronic pain, provides a potential explanation for conflicting data sets published in the field^10^.

## Material and methods

### Animals and housing

The study was performed in accordance with the Danish legislation (Law no. 474 of May 15th, 2014 and Order no. 12 of 07/01/2016) regulating experiments on animals, and in compliance with the European Directive 2010/63/EU. Experimental protocols for the different testing modalities at H. Lundbeck A/S were approved by The Animal Experiments Inspectorate in Denmark.

A total of 18 rats per strain (Lewis (LEW) and Wistar Kyoto (WKY) from Harlan / Envigo UK; F344/IcoCrl from Charles River, Italy; F344/DuCrl and Crl:CD(SD) from Charles River Laboratories, Germany) were ordered to arrive in house at 6 weeks of age. All animals were housed in pairs with a partner from the same strain and surgical group in transparent Tecniplast polycarbonate macrolone type III high open cages (42.5 * 26.6 * 18.5 cm) from Scanbur, Denmark, from arrival and until they reached a body weight of approximately 400 g per rat. At this point all pairs of rats were moved to larger type IV high open cages (59.5*38.0*20 cm). All cages were equipped with environmental enrichment consisting of aspen wood chewing blocks (S-Bricks from Tapvei, Estonia), paper-wool shavings (LBS Biotechnology, UK) for nesting material, and red Rat Retreats (Bio-Serv, Flemington, US) for hiding. For bedding, aspen chips (Tapvei, Estonia) were used. Cages were changed twice a week, but never on testing days. Food (Altromin 1324, Brogaarden, Denmark). Acidified water (pH: 3.6 ± 0.5) was available ad libitum in two bottles per cage at all times, and were changed on a weekly basis. The light–dark cycle was 12:12 h with lights on from 06.00 h. The room-temperature was set to 20 ± 2 °C and the relative humidity was 55 ± 10%.

### Study design

For pragmatic purposes test subjects were divided in two cohorts each including animals from all strains and surgical groups. The two cohorts were subjected to surgery on consecutive days enabling behavioural testing to be performed between 08.00–15.00 h during the light cycle on alternate days. Only sucrose preference testing (SPT), and sampling for fecal corticosterone measurement (FCM) were performed simultaneously for the two cohorts. In total, eight rats of each strain were exposed to sham-surgery, and ten rats to SNI-surgery.

**Figure 8:**
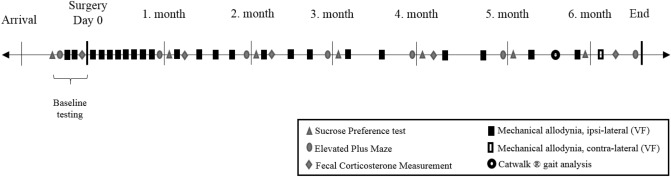
Timeline of behavioural tests and corticosterone sampling performed during the study.

After arrival from the vendors, the rats acclimatized to the surroundings for 12 days prior to initiation of baseline testing. They remained in the same room for the majority of the study, but were moved to a different room in the same building inside the facility for elevated plus maze (EPM) and CatWalk testing. When this occurred they were always moved at least 24–48 h prior to behavioural testing, and then moved back to the primary lab immediately after the end of the test day. The overall timeline for the study is presented in Fig. 8. Baseline tests were performed prior to surgery for SPT (Day − 15), EPM (Day − 12), von Frey (Day − 10 and − 7), gut microbiota (Day − 7) (results not included in current manuscript) and FCM (Day − 1). The day of surgery was considered as ‘Day 0′. After surgery EPM and SPT were measured once a month for 6 months, while FCM and gut microbiota was determined at 1, 2, 4, and 6 months post nerve injury (gut microbiota results are planned to be presented together with additional experiments in a separate publication). Mechanical allodynia of the ipsi-lateral paw was measured more frequently (Day 3, 6, 9, 13, 16, 20, 23, 27, 34, 41, 48, 55, 65, 76, 84, 97, 111, 128, 146, 160, 177), while the mechanical threshold of the contra-lateral paw was only measured at Day 182. CatWalk gait analysis was performed once only between Days 168–171 after surgery. At the end of the experiment (Days 188–194 post surgery) all animals were euthanized by decapitation without prior sedation, and tissues were extracted for western blotting (WB) analysis. In addition, pituitary and adrenal glands were collected and weighed.

Although animals were pair-housed from the beginning of the experiment, three SD rats, one F344/DU rat and one F344/ICO rat from SNI-groups were euthanized in accordance with predefined humane endpoints; their partners were single-housed for the remainder of the study. All rats were euthanized due to biting of the injured paw, which occasionally occurs after SNI-injury^55^, except for one SD rat which was euthanized due to an incidental injury unrelated to the experiment.

### Experimental procedures

All experiments were performed by the same experienced female experimenter throughout the study. It was not possible to blind the experimenter to strains and surgeries, as the strains behaved markedly different when being handled, and SNI-surgery leads to a characteristic pronation of the affected paw^56^.

#### Spared nerve injury

The surgical procedure was based on the model described by Decosterd and Woolf^112^, and the same experienced surgeon performed all surgeries as previously reported by our group^22,113^. Anesthesia was induced with 5.0–5.5% sevoflurane (SEVOrane, AbbVie Inc.) delivered in a mixture of 70% O_2_ and 30% N_2_O in a Plexiglas induction chamber, and maintained via a face mask with 2–3% sevoflurane in the same O_2_/N_2_O mixture. Anesthesia was monitored regularly by observing respiration and testing the hind paw withdrawal reflex. Each rat was administered a single dose of buprenorphine (Temgesic, 0.03 mg/kg) and amoxicillintrihydrate (Noromox Prolongatum Vet, 150 mg/kg). Thereafter, the fur was shaved on the lateral surface of the left thigh and the area was swabbed with chlorhexidine to secure aseptic conditions. A longitudinal incision was made through the skin caudal to the femur, and the underlying musculature was opened using blunt dissection to reveal the sciatic nerve and the three terminal branches; the sural, common peroneal and tibial nerves. A gentle pinch with forceps was performed on the common peroneal and tibial nerves before ligation to verify that they were the intended nerves^55^. The common peroneal and tibial nerves were ligated together using 5–0 Prolene ligatures (Jørgen Kruuse A/S, Denmark), and sectioned distally to the ligation, removing approximately 2 mm of the distal nerve stump. The spared sural nerve was left intact. The musculature was reapproximated and the skin closed with tissue glue (3 M Vetbond, Jørgen Kruuse A/S, Denmark). Sham-surgery consisted of the same steps, but without opening of the muscle layers or any contact with the sciatic nerve. Animals were examined routinely for signs of distress, wound dehiscence or wound infection throughout the study in accordance with welfare assessment^113,114^.

#### Mechanical allodynia

Low intensity mechanical sensitivity was assessed using a series of calibrated von Frey monofilaments (0.4, 0.6, 1.0, 1.4, 2.0, 4.0, 6.0, 8.0, 10.0, 15.0, 26.0 g; North Coast Medical, Inc., Morgan Hill), similarly to previously descibed^22^. Animals were placed in individual plexiglas (10.8*13.8*17.0 cm) enclosures on an elevated wire grid located in the same room in which they were routinely housed. They were given approximately 15 min to acclimate to the enclosure and the experimenter’s presence prior to stimulation of the lateral plantar surface of the hind paw, at different anatomical locations in the area innervated by the intact sural nerve. To initiate testing a filament with a bending force of 4.0 g was first applied to the hind paw with uniform pressure for 5 s. A brisk withdrawal was considered a positive response whereupon the next lower filament in the series was applied. In the absence of a positive response the neighboring higher filament was applied. After the first change in response-pattern, indicating the threshold, 4 additional applications were performed; when no response, the next filament with a higher force was tested, and when positive response, the next lower force filament was tested. The 50% threshold was determined by the following equation: 50% threshold (g) = 10^log(last filament)+k*0.3^. The constant, *k*, was found in the table by Dixon^115^, and determined by the response-pattern.

#### Elevated plus maze

To assess anxiety-like behaviour an EPM was used as described and validated previously^22,116^. The maze consisted of four arms (45*10 cm) arranged in a cross-like disposition. Two opposite arms were open, and the other two were equipped with 50 cm high walls on each side for enclosement. All were connected by a central 10*10 cm square. The surface of the maze was covered in a grid-safe black rubber flooring to increase contrast between the white test-subject and the background. Recording was performed for 5 min per rat by use of a camera placed above the maze, and movement between zones was tracked by use of EthoVision XT9 (Noldus Information Technology). The maze was wiped with 70% ethanol between each animal to minimize the influence of odours between rats. The EPM was placed in a separate room close to the housing room, with light-intensity set to; centre: 276 lx, open: 283–388 lx (near centre—distal end of open arm) and closed: 19–82 lx (distal end of closed arm – near centre).

#### Sucrose preference test

In order to assess depressive-like behaviour, the sucrose preference test was included as a measure of anhedonia^89^. Throughout the study, each cage was fitted with two water bottles, in order to have the rats accustomed to drinking from both bottles / sides of the cage. The SPT was performed on a cage-level in the normal environment with no other testing occurring during those days. When performing the test two fresh bottles were placed in the cage, one of which included sucrose (2%) dissolved in normal acidified drinking water. Bottles were weighed and sucrose- vs. water-consumption was measured during 2*24 h tests. The bottles were weighed after the first 24 h and swapped to the opposite side of the cage, in order to compensate for possible differences in preference for drinking from one side. The preference was then calculated and presented as “% sucrose preference”, but also presented as amount of sucrose consumed (sucrose (g) / body weight (g)), as recent studies suggested that this parameter could be a relevant alternative^33^.

#### CatWalk gait analysis

Analysis of voluntary movement and gait pattern was performed using the CatWalk XT 10.0 system (Noldus Information Technology)^60^. Briefly, green light was internally reflected into a glass plate, on which an enclosed corridor was fixed, with red backlight above the corridor. A video-camera was mounted underneath the setup and recorded the paw prints being lit up by the green light when paws were in contact with the glass plate as the rat walked along the corridor. A run was regarded as compliant when the rat entered in one end of the corridor, and moved fluently across the plate towards the exit in the other end of the corridor, with a running duration below 12 s and a maximum variation below 75%. Three compliant runs were recorded for each animal, with no previous training/habituation or food-deprivation.

#### Measurement of fecal corticosterone and immunoreactive corticosterone metabolites

HPA-axis activation was gauged by quantifying fecal corticosterone and immunoreactive corticosterone metabolites (FCM). The majority (80–87%) of the circulating corticosterone is excreted in feces after undergoing metabolism in the liver^117–119^, and levels of FCM reflects an average of the preceding corticosterone levels in the blood with approximately 8–12 h delay^120,121^. On the days selected for measurement of FCM, all bedding was changed in the morning thereby covering the previous 24 h period. Fecal pellets were collected from the bedding and kept at − 20 °C until analysis similar to previously reported^121,122^, with some modifications regarding evaporation^113^. In brief, all pellets from the specific cage and day were submerged in 96% ethanol (~ 5 ml/g feces) overnight at room temperature on a shaking table. Samples were then homogenized in a BagMixer (BagMixer 400CC, Interscience, Saint Nom, France), then filtered free of fecal material and centrifuged (2*20 min at 2,000 rpm, and 1*15 min at 10,000 rpm), and 1 ml of the supernatant was stored at − 20 °C. For analysis, 300 μl of the supernatant was processed in an evaporator (Genevac EZ-2 Personal Evaporator, Stone Ridge, NY, USA) for approximately 2 h. Next, 300 μl PBS was added to each sample along with three to four 2 mm solid-glass beads (Sigma-Aldrich, St Louis, MO, USA) and placed on a shaking table for 2 h prior to quantification. The samples were then quantified using the DRG-Diagnostics Corticosterone ELISA kit (EIA-4164; DRG Instruments GmBH, Maburg, Germany) according to the manufacturer’s instructions and as previously described^113,122^. The ELISA-kit has been shown to have a cross-reactivity with other steroids and corticosterone metabolites (7.4% with progesterone, 3.4% with deoxycorticosterone, 1.6% with 11-dehydrocorticosterone, 0.3% with cortisol and pregnenolone and < 0.1% with other steroids), and have previously been validated and used for measurements of fecal corticosterone metabolites in rodents^113,122,123^.

#### Western Blot analysis

At the end of the study, rats were euthanized by decapitation without prior sedation to enable collection of spinal cords and brains for quantification of mu- and kappa-opioid receptor (MOP and KOP respectively) expression by WB analysis. The spinal cord was collected via hydraulic extrusion using a previously published protocol^124^. Thereafter, the lumbar enlargement of the spinal cord was dissected into dorsal/ventral, ipsi- and contra-lateral quadrants using macroscopic anatomical landmarks. Following decapitation, the skin was removed from the skull, and a small pair of surgical scissors was inserted into the foramen magnum to carefully break and cut the posterior part of the cranium. This enabled the occipital, interparietal, parietal and frontal bone plates of the skull to be removed and with the brain exposed the dura mater was subsequently removed. A dissecting spatula was carefully slid between the bone and lateral part of the brain-hemispheres, to carefully free the brain from the skull, and the olfactory, optical and trigeminal nerve attachments were gently cut to completely free the brain. While kept on ice, the brain was dissected using macroscopically visible anatomical landmarks. We used free hand dissection with a straight edge razor blade and forceps for the majority of the areas, except where noted otherwise. We did not use definitive scales or ‘rodent brain matrix’ devices, since the different strains were of overall different sizes, which could naturally affect the dimensions of the brain. First, the hypothalamus was localized and excised with the aid of forceps and a blade, so that the most distal part of the hypothalamus was collected. Next after removing the olfactory bulb in the anterior part of the brain and using a sharp razor blade, a coronal section was made including the most anterior part of the prefrontal cortex (PFC). Next, a latero-medial transverse section was made with the straight edge razor blade including predominantly amygdala, but likely also including some piriform cortex. As the dissection was based on gross/macroscopic anatomy, the amygdala is hereafter termed ‘amygdaloid complex’, and has also been referred to as ‘amygdaloid cortex’ in previous publications using the same dissection-technique^125,126^. Next, the periaqueductal gray (PAG) in the midbrain was collected, which included trimming off the surrounding white matter. Finally, the part of the brain-stem ventral to the cerebellum was isolated, and a 2 mm diameter cylindrical biopsy puncher (Harvard Apparatus, UK) was used to excise the rostral ventromedial medulla (RVM). All tissues were weighed, snap-frozen on dry ice, and stored at − 80 °C. Due to time-limitations, analysis was only performed on samples from 8 subjects from SNI- and sham-groups of the F344/DU, F344/ICO and LEW strains. These strains were selected because they are commonly compared, showed prominent behavioural differences during the study, and have been found to respond differently to opioid agonist in the presence and absence of injury^26^.

Protein extraction from the different brain areas was performed according to methods previously described^23^. 36 µg protein from each sample was separated using a 12% SDS–polyacrylamide gel (SDS-PAGE) electrophoresis at a constant voltage of 120 V for approximately 2 h. Each gel included samples from each strain and group, along with a control-sample for comparison between gels/blots. Separated proteins were then electro-blotted to a Nitrocellulose Blotting Membrane (GE Healthcare Life Science, catalogue No 10600003) at 100 V for 50 min. The nitrocellulose membranes were then blocked in 5% non-fat dry milk dissolved in 0.05% Tris-buffered saline with Tween 20 (TBS-T) for 1 h at room temperature (RT), and incubated with antibody diluted in 5% milk / TBS-T. For each brain-area/sample a set of gels and blots were made for incubation with different primary antibodies using different protocols, while the protocol for incubation with secondary antibodies were similar for all blots. Opioid-receptor blots were first incubated with primary MOP-antibody (Anti-Mu Opioid Receptor antibody ab10275, Abcam) and β-actin (1:10.000, Sigma Aldrich, Ireland) for 2 h at RT and overnight at 4 °C. After incubation with primary antibodies, blots were subjected to 5*5 min washes in 0.05% TBS-T, and incubated with secondary antibody solution containing IRDye 800CW conjugated goat anti-rabbit (926–32,211) for binding with MOP-primary antibodies, and IRDye 680LT Goat anti-Mouse (926–68,020) (LI-COR Biosciences Abingdon Park, Oxford UK) for binding with β-actin, in 1:10.000 dilution in 1% milk / TBS-T for 2 h covered in tin-foil. Then another 5*5 min wash with TBS-T, before blots were scanned using a LI-COR Odyssey imager, and afterwards analysis was performed using Image Studio Software (LI-COR Bioscience). After incubation with the secondary antibody and scanning of blots, they were stripped and re-incubated with MOP according to the same protocol. Depending on brain area analysed, some blots were then stripped for antibodies, and incubated with KOP (1:1000, Rabbit (polyclonal) κ-Opioid Receptor Antibody, Invitrogen (44302G)) for 1 h at RT and overnight at 4 °C. Note that as the hypothalamus-blots were the first immunoblots performed in the series, we first incubated them with the MOP-antibody alone, and next incubated with β-actin after stripping the blots for MOP-antibody. This was done for all hypothalamus blots simultaneously, and to verify that there was no overlap of the β-actin and MOP-bands. For the remaining tissues tested, however, we included both MOP and β-actin in the same round of staining, and for the KOP-blotting, the β-actin-analysis from the MOP-incubation was reused as reference for the individual samples. MOP-bands were determined at ~ 53 kDa, KOP at 43 kDa, and β-actin at ~ 42 kDa, as specified in the antibody-supplier information, and analysed using Image Studio Software (LI-COR Bioscience). For the KOP-antibody, for some immunoblots, there was an occurrence of an additional band at ~ 25 kDa, which was not specified in the antibody supplier information. Although using protease inhibitor in the lysis buffer, this band is likely representing a degraded form of the receptor-protein, which have been presented before using both KOP- and MOP-antibodies^127^. This additional band was not analysed.

### Data analysis and statistics

Animals were randomly allocated to surgical groups in co-housed pairs. No specific power analysis was performed in the current study. However, group sizes were calculated based on previous in house SNI experiments in which power analysis was applied. Statistical analysis was performed using GraphPad Prism version 5 (GraphPad Software, Inc., La Jolla, CA, USA) for the majority of the analysis (analysis of variance [ANOVA]). Analysis of co-variance [ANCOVA] was performed in IBM SPSS Statistics, version 24 (IBM Corp.). *P* < 0.05 was considered statistically significant.

## Supplementary information

Supplementary Information.

## Data Availability

The datasets generated and analyzed during the current study are available from the corresponding author on reasonable request.

## References

[1] Baron R (2017). Peripheral neuropathic pain: a mechanism-related organizing principle based on sensory profiles. Pain.

[2] Demant DT (2014). The effect of oxcarbazepine in peripheral neuropathic pain depends on pain phenotype: a randomised, double-blind, placebo-controlled phenotype-stratified study. Pain.

[3] Rice ASC, Finnerup NB, Kemp HI, Currie GL, Baron R (2018). Sensory profiling in animal models of neuropathic pain: a call for back-translation. Pain.

[4] Asmundson GJ, Katz J (2009). Understanding the co-occurrence of anxiety disorders and chronic pain: state-of-the-art. Depress Anxiety.

[5] Bair MJ, Robinson RL, Katon W, Kroenke K (2003). Depression and pain comorbidity: a literature review. Arch. Intern. Med..

[6] Gold PW, Machado-Vieira R, Pavlatou MG (2015). Clinical and biochemical manifestations of depression: relation to the neurobiology of stress. Neural Plast..

[7] Jennings EM, Okine BN, Roche M, Finn DP (2014). Stress-induced hyperalgesia. Prog. Neurobiol..

[8] Ferdousi M, Finn DP (2018). Stress-induced modulation of pain: role of the endogenous opioid system. Prog. Brain Res..

[9] Radat F, Margot-Duclot A, Attal N (2013). Psychiatric co-morbidities in patients with chronic peripheral neuropathic pain: a multicentre cohort study. Eur. J. Pain.

[10] Yalcin I, Barthas F, Barrot M (2014). Emotional consequences of neuropathic pain: insight from preclinical studies. Neurosci. Biobehav. Rev..

[11] Leite-Almeida H (2009). The impact of age on emotional and cognitive behaviours triggered by experimental neuropathy in rats. Pain.

[12] Roeska K, Doods H, Arndt K, Treede RD, Ceci A (2008). Anxiety-like behaviour in rats with mononeuropathy is reduced by the analgesic drugs morphine and gabapentin. Pain.

[13] Leite-Almeida H (2012). Differential effects of left/right neuropathy on rats' anxiety and cognitive behavior. Pain.

[14] Seminowicz DA (2009). MRI structural brain changes associated with sensory and emotional function in a rat model of long-term neuropathic pain. Neuroimage.

[15] Low LA (2012). Nerve injury causes long-term attentional deficits in rats. Neurosci. Lett..

[16] Hubbard CS (2015). Behavioral, metabolic and functional brain changes in a rat model of chronic neuropathic pain: a longitudinal MRI study. Neuroimage.

[17] Lariviere WR, Mogil JS (2010). The genetics of pain and analgesia in laboratory animals. Methods Mol. Biol..

[18] van der Staay FJ, Schuurman T, van Reenen CG, Korte SM (2009). Emotional reactivity and cognitive performance in aversively motivated tasks: a comparison between four rat strains. Behav. Brain Funct..

[19] Rode F (2007). The importance of genetic background on pain behaviours and pharmacological sensitivity in the rat spared serve injury model of peripheral neuropathic pain. Eur. J. Pharmacol..

[20] Pothion S, Bizot JC, Trovero F, Belzung C (2004). Strain differences in sucrose preference and in the consequences of unpredictable chronic mild stress. Behav. Brain Res..

[21] Ramos A, Berton O, Mormede P, Chaouloff F (1997). A multiple-test study of anxiety-related behaviours in six inbred rat strains. Behav. Brain Res..

[22] Hestehave S, Abelson KS, Bronnum Pedersen T, Munro G (2019). Stress sensitivity and cutaneous sensory thresholds before and after neuropathic injury in various inbred and outbred rat strains. Behav. Brain Res..

[23] Rea K (2014). Impaired endocannabinoid signalling in the rostral ventromedial medulla underpins genotype-dependent hyper-responsivity to noxious stimuli. Pain.

[24] Madasu MK (2016). Genotype-dependent responsivity to inflammatory pain: a role for TRPV1 in the periaqueductal grey. Pharmacol. Res..

[25] Kremer M, Becker LJ, Barrot M, Yalcin I (2020). How to study anxiety and depression in rodent models of chronic pain?. Eur. J. Neurosci..

[26] Hestehave S, Abelson KSP, Brønnum Pedersen T, Munro G (2019). The analgesic efficacy of morphine varies with rat strain and experimental pain model: implications for target validation efforts in pain drug discovery. Eur. J. Pain.

[27] Chiba S (2012). Chronic restraint stress causes anxiety- and depression-like behaviors, downregulates glucocorticoid receptor expression, and attenuates glutamate release induced by brain-derived neurotrophic factor in the prefrontal cortex. Prog. Neuropsychopharmacol. Biol. Psychiatry.

[28] Blackburn-Munro G (2004). Hypothalamo-pituitary-adrenal axis dysfunction as a contributory factor to chronic pain and depression. Curr. Pain Headache Rep..

[29] da Silva Torres IL (2003). Long-lasting delayed hyperalgesia after chronic restraint stress in rats-effect of morphine administration. Neurosci. Res..

[30] Caspi A (2003). Influence of life stress on depression: moderation by a polymorphism in the 5-HTT gene. Science.

[31] Narita M (2006). Chronic pain induces anxiety with concomitant changes in opioidergic function in the amygdala. Neuropsychopharmacology.

[32] Nation KM (2018). Lateralized kappa opioid receptor signaling from the amygdala central nucleus promotes stress-induced functional pain. Pain.

[33] Hakim JD, Chami J, Keay KA (2020). mu-Opioid and dopamine-D2 receptor expression in the nucleus accumbens of male Sprague-Dawley rats whose sucrose consumption, but not preference, decreases after nerve injury. Behav. Brain Res..

[34] Thompson SJ (2018). Chronic neuropathic pain reduces opioid receptor availability with associated anhedonia in rat. Pain.

[35] Humo M, Lu H, Yalcin I (2019). The molecular neurobiology of chronic pain–induced depression. Cell Tissue Res..

[36] Pitzer C, Kuner R, Tappe-Theodor A (2016). EXPRESS: voluntary and evoked behavioral correlates in neuropathic pain states under different housing conditions. Mol. Pain.

[37] Shepherd AJ, Mohapatra DP (2018). Pharmacological validation of voluntary gait and mechanical sensitivity assays associated with inflammatory and neuropathic pain in mice. Neuropharmacology.

[38] Matsuda K (2016). Swing time ratio, a new parameter of gait disturbance, for the evaluation of the severity of neuropathic pain in a rat model of partial sciatic nerve ligation. J. Pharmacol. Toxicol. Methods.

[39] Mogil JS (2010). Hypolocomotion, asymmetrically directed behaviors (licking, lifting, flinching, and shaking) and dynamic weight bearing (gait) changes are not measures of neuropathic pain in mice. Mol. Pain.

[40] Wu HH, Wang S (2010). Strain differences in the chronic mild stress animal model of depression. Behav. Brain Res..

[41] Berge O-G (2011). Predictive validity of behavioural animal models for chronic pain. Br. J. Pharmacol..

[42] Mogil JS (2009). Animal models of pain: progress and challenges. Nat. Rev. Neurosci..

[43] Blackburn-Munro G (2004). Pain-like behaviours in animals—how human are they?. Trends Pharmacol. Sci..

[44] Zeng Q (2008). Exacerbated mechanical allodynia in rats with depression-like behavior. Brain Res..

[45] del Rey A (2011). Chronic neuropathic pain-like behavior correlates with IL-1beta expression and disrupts cytokine interactions in the hippocampus. Pain.

[46] Yoon YW, Lee DH, Lee BH, Chung K, Chung JM (1999). Different strains and substrains of rats show different levels of neuropathic pain behaviors. Exp. Brain Res..

[47] Lovell JA, Stuesse SL, Cruce WL, Crisp T (2000). Strain differences in neuropathic hyperalgesia. Pharmacol. Biochem. Behav..

[48] Le Coz GM, Fiatte C, Anton F, Hanesch U (2014). Differential neuropathic pain sensitivity and expression of spinal mediators in Lewis and Fischer 344 rats. BMC Neurosci..

[49] Herradon G (2007). Changes in BDNF gene expression correlate with rat strain differences in neuropathic pain. Neurosci. Lett..

[50] Glowa JR, Hansen CT (1994). Differences in response to an acoustic startle stimulus among forty-six rat strains. Behav. Genet..

[51] Stohr T, Szuran T, Pliska V, Feldon J (1999). Behavioural and hormonal differences between two Lewis rat lines. Behav. Brain Res..

[52] Zhang-James Y, Middleton FA, Faraone SV (2013). Genetic architecture of Wistar–Kyoto rat and spontaneously hypertensive rat substrains from different sources. Physiol. Genom..

[53] Kristensen PJ (2017). Vendor-derived differences in injury-induced pain phenotype and pharmacology of Sprague-Dawley rats: does it matter?. Eur. J. Pain.

[54] Rigaud M (2008). Species and strain differences in rodent sciatic nerve anatomy: implications for studies of neuropathic pain. Pain.

[55] Pertin M, Gosselin RD, Decosterd I (2012). The spared nerve injury model of neuropathic pain. Methods Mol. Biol..

[56] Lau W (2013). A back translation of pregabalin and carbamazepine against evoked and non-evoked endpoints in the rat spared nerve injury model of neuropathic pain. Neuropharmacology.

[57] Rolke R (2006). Quantitative sensory testing in the german research network on neuropathic pain (DFNS): standardized protocol and reference values. Pain.

[58] Mogil JS, Crager SE (2004). What should we be measuring in behavioral studies of chronic pain in animals?. Pain.

[59] Clark JD (2016). Preclinical pain research: can we do better?. Anesthesiology.

[60] Vrinten DH, Hamers FF (2003). 'CatWalk' automated quantitative gait analysis as a novel method to assess mechanical allodynia in the rat; a comparison with von Frey testing. Pain.

[61] Deumens R, Jaken RJ, Marcus MA, Joosten EA (2007). The CatWalk gait analysis in assessment of both dynamic and static gait changes after adult rat sciatic nerve resection. J. Neurosci. Methods.

[62] Alba-Delgado C (2013). Chronic pain leads to concomitant noradrenergic impairment and mood disorders. Biol. Psychiatry.

[63] Benbouzid M (2008). Sciatic nerve cuffing in mice: a model of sustained neuropathic pain. Eur. J. Pain.

[64] Goncalves L (2008). Neuropathic pain is associated with depressive behaviour and induces neuroplasticity in the amygdala of the rat. Exp. Neurol..

[65] Suzuki T (2007). Experimental neuropathy in mice is associated with delayed behavioral changes related to anxiety and depression. Anesth. Analgesia.

[66] Yalcin I (2011). A time-dependent history of mood disorders in a murine model of neuropathic pain. Biol. Psychiatry.

[67] Matsuzawa-Yanagida K (2008). Usefulness of antidepressants for improving the neuropathic pain-like state and pain-induced anxiety through actions at different brain sites. Neuropsychopharmacology.

[68] Fukuhara K (2012). Intracerebroventricular 4-methylcatechol (4-MC) ameliorates chronic pain associated with depression-like behavior via induction of brain-derived neurotrophic factor (BDNF). Cell Mol. Neurobiol..

[69] Sellmeijer J (2018). Hyperactivity of anterior cingulate cortex areas 24a/24b drives chronic pain-induced anxiodepressive-like consequences. J. Neurosci. Off. J. Soc. Neurosci..

[70] Kontinen VK, Kauppila T, Paananen S, Pertovaara A, Kalso E (1999). Behavioural measures of depression and anxiety in rats with spinal nerve ligation-induced neuropathy. Pain.

[71] Hasnie FS, Wallace VC, Hefner K, Holmes A, Rice AS (2007). Mechanical and cold hypersensitivity in nerve-injured C57BL/6J mice is not associated with fear-avoidance- and depression-related behaviour. Br. J. Anaesth..

[72] Kodama D, Ono H, Tanabe M (2011). Increased hippocampal glycine uptake and cognitive dysfunction after peripheral nerve injury. Pain.

[73] Urban R, Scherrer G, Goulding EH, Tecott LH, Basbaum AI (2011). Behavioral indices of ongoing pain are largely unchanged in male mice with tissue or nerve injury-induced mechanical hypersensitivity. Pain.

[74] Pitzer C, La Porta C, Treede R-D, Tappe-Theodor A (2019). Inflammatory and neuropathic pain conditions do not primarily evoke anxiety-like behaviours in C57BL/6 mice. Eur. J. Pain.

[75] Okun A (2016). Hedonic and motivational responses to food reward are unchanged in rats with neuropathic pain. Pain.

[76] Sieberg CB (2018). Neuropathic pain drives anxiety behavior in mice, results consistent with anxiety levels in diabetic neuropathy patients. PAIN Rep..

[77] Dimitrov EL, Tsuda MC, Cameron HA, Usdin TB (2014). Anxiety- and depression-like behavior and impaired neurogenesis evoked by peripheral neuropathy persist following resolution of prolonged tactile hypersensitivity. J. Neurosci..

[78] Pare WP (1994). Open field, learned helplessness, conditioned defensive burying, and forced-swim tests in WKY rats. Physiol. Behav..

[79] Shepard JD, Myers DA (2008). Strain differences in anxiety-like behavior: association with corticotropin-releasing factor. Behav. Brain Res..

[80] Pardon MC (2002). Stress reactivity of the brain noradrenergic system in three rat strains differing in their neuroendocrine and behavioral responses to stress: implications for susceptibility to stress-related neuropsychiatric disorders. Neuroscience.

[81] Solberg LC, Olson SL, Turek FW, Redei E (2001). Altered hormone levels and circadian rhythm of activity in the WKY rat, a putative animal model of depression. Am. J. Physiol. Regul. Integr. Comp. Physiol..

[82] Pare WP (1992). The performance of WKY rats on three tests of emotional behavior. Physiol. Behav..

[83] Dhabhar FS, McEwen BS, Spencer RL (1993). Stress response, adrenal steroid receptor levels and corticosteroid-binding globulin levels–a comparison between Sprague–Dawley, Fischer 344 and Lewis rats. Brain Res..

[84] Sternberg EM (1989). A central nervous system defect in biosynthesis of corticotropin-releasing hormone is associated with susceptibility to streptococcal cell wall-induced arthritis in Lewis rats. Proc. Natl. Acad. Sci. USA.

[85] Gomez F, Lahmame A, de Kloet ER, Armario A (1996). Hypothalamic-pituitary-adrenal response to chronic stress in five inbred rat strains: differential responses are mainly located at the adrenocortical level. Neuroendocrinology.

[86] Ramos A (2002). Evaluation of Lewis and SHR rat strains as a genetic model for the study of anxiety and pain. Behav. Brain Res..

[87] Hinojosa FR (2006). Evaluation of two genetic animal models in behavioral tests of anxiety and depression. Behav. Brain Res..

[88] Guimaraes MR (2019). Evidence for lack of direct causality between pain and affective disturbances in a rat peripheral neuropathy model. Genes Brain Behav..

[89] Willner P, Towell A, Sampson D, Sophokleous S, Muscat R (1987). Reduction of sucrose preference by chronic unpredictable mild stress, and its restoration by a tricyclic antidepressant. Psychopharmacology.

[90] Mutso AA (2012). Abnormalities in hippocampal functioning with persistent pain. J. Neurosci..

[91] Goffer Y (2013). Calcium-permeable AMPA receptors in the nucleus accumbens regulate depression-like behaviors in the chronic neuropathic pain state. J. Neurosci..

[92] Racz I, Nent E, Erxlebe E, Zimmer A (2015). CB1 receptors modulate affective behaviour induced by neuropathic pain. Brain Res. Bull..

[93] Dellarole A (2014). Neuropathic pain-induced depressive-like behavior and hippocampal neurogenesis and plasticity are dependent on TNFR1 signaling. Brain Behav. Immun..

[94] Willner P (1997). Validity, reliability and utility of the chronic mild stress model of depression: a 10-year review and evaluation. Psychopharmacology.

[95] Bushnell MC (2015). Effect of environment on the long-term consequences of chronic pain. Pain.

[96] Wu SX (2010). The synaptic connectivity that underlies the noxious transmission and modulation within the superficial dorsal horn of the spinal cord. Prog. Neurobiol..

[97] Burke NN, Finn DP, Roche M (2015). Neuroinflammatory mechanisms linking pain and depression. Mod.

[98] Bomholt SF, Mikkelsen JD, Blackburn-Munro G (2005). Normal hypothalamo-pituitary-adrenal axis function in a rat model of peripheral neuropathic pain. Brain Res..

[99] Ulrich-Lai YM (2006). Limbic and HPA axis function in an animal model of chronic neuropathic pain. Physiol. Behav..

[100] Grota LJ, Bienen T, Felten DL (1997). Corticosterone responses of adult Lewis and Fischer rats. J. Neuroimmunol..

[101] Lutz PE, Kieffer BL (2013). Opioid receptors: distinct roles in mood disorders. Trends Neurosci..

[102] Ossipov MH, Dussor GO, Porreca F (2010). Central modulation of pain. J. Clin. Investig..

[103] Ossipov MH, Morimura K, Porreca F (2014). Descending pain modulation and chronification of pain. Curr. Opin. Support. Palliat. Care.

[104] De Felice M (2011). Engagement of descending inhibition from the rostral ventromedial medulla protects against chronic neuropathic pain. Pain.

[105] Jennings EM, Okine BN, Olango WM, Roche M, Finn DP (2016). Repeated forced swim stress differentially affects formalin-evoked nociceptive behaviour and the endocannabinoid system in stress normo-responsive and stress hyper-responsive rat strains. Prog. Neuropsychopharmacol. Biol. Psychiatry.

[106] Okine BN (2017). Characterisation of peroxisome proliferator-activated receptor signalling in the midbrain periaqueductal grey of rats genetically prone to heightened stress, negative affect and hyperalgesia. Brain Res..

[107] Lim G, Sung B, Ji RR, Mao J (2003). Upregulation of spinal cannabinoid-1-receptors following nerve injury enhances the effects of Win 55,212–2 on neuropathic pain behaviors in rats. Pain.

[108] Zubieta JK (2001). Regional mu opioid receptor regulation of sensory and affective dimensions of pain. Science.

[109] Goncalves L, Dickenson AH (2012). Asymmetric time-dependent activation of right central amygdala neurones in rats with peripheral neuropathy and pregabalin modulation. Eur. J. Neurosci..

[110] Spampinato MV, Wood JN, De Simone V, Grafman J (2009). Neural correlates of anxiety in healthy volunteers: a voxel-based morphometry study. J. Neuropsychiatry Clin. Neurosci..

[111] Sanchez-Cardoso P (2007). Modulation of the endogenous opioid system after morphine self-administration and during its extinction: a study in Lewis and Fischer 344 rats. Neuropharmacology.

[112] Decosterd I, Woolf CJ (2000). Spared nerve injury: an animal model of persistent peripheral neuropathic pain. Pain.

[113] Hestehave S (2017). Is there a reasonable excuse for not providing post-operative analgesia when using animal models of peripheral neuropathic pain for research purposes?. PLoS ONE.

[114] Hampshire VA, Davis JA, McNickle CA, Williams L, Eskildson H (2001). Retrospective comparison of rat recovery weights using inhalation and injectable anaesthetics, nutritional and fluid supplementation for right unilateral neurosurgical lesioning. Lab. Anim..

[115] Dixon WJ (1980). Efficient analysis of experimental observations. Annu. Rev. Pharmacol. Toxicol..

[116] Pellow S, Chopin P, File SE, Briley M (1985). Validation of open:closed arm entries in an elevated plus-maze as a measure of anxiety in the rat. J. Neurosci. Methods.

[117] Bamberg E, Palme R, Meingassner JG (2001). Excretion of corticosteroid metabolites in urine and faeces of rats. Lab. Anim..

[118] Royo F, Bjork N, Carlsson HE, Mayo S, Hau J (2004). Impact of chronic catheterization and automated blood sampling (Accusampler) on serum corticosterone and fecal immunoreactive corticosterone metabolites and immunoglobulin A in male rats. J. Endocrinol..

[119] Touma C, Palme R (2005). Measuring fecal glucocorticoid metabolites in mammals and birds: the importance of validation. Ann. N.Y. Acad. Sci..

[120] Siswanto H, Hau J, Carlsson HE, Goldkuhl R, Abelson KS (2008). Corticosterone concentrations in blood and excretion in faeces after ACTH administration in male Sprague–Dawley rats. Vivo.

[121] Sundbom R, Jacobsen KR, Kalliokoski O, Hau J, Abelson KS (2011). Post-operative corticosterone levels in plasma and feces of mice subjected to permanent catheterization and automated blood sampling. Vivo.

[122] Kalliokoski O, Jacobsen KR, Teilmann AC, Hau J, Abelson KS (2012). Quantitative effects of diet on fecal corticosterone metabolites in two strains of laboratory mice. Vivo.

[123] Abelson KS, Kalliokoski O, Teilmann AC, Hau J (2016). Applicability of commercially available ELISA Kits for the quantification of faecal immunoreactive corticosterone metabolites in mice. Vivo.

[124] Richner M, Jager SB, Siupka P, Vaegter CB (2017). Hydraulic extrusion of the spinal cord and isolation of dorsal root ganglia in rodents. JoVE.

[125] Roche M, O'Connor E, Diskin C, Finn DP (2007). The effect of CB1 receptor antagonism in the right basolateral amygdala on conditioned fear and associated analgesia in rats. Eur. J. Neurosci..

[126] Smith KL, Ford GK, Jessop DS, Finn DP (2013). Behavioural, neurochemical and neuroendocrine effects of the endogenous β-carboline harmane in fear-conditioned rats. J. Psychopharmacol..

[127] Ujcikova H (2017). Determination of μ-, δ- and κ-opioid receptors in forebrain cortex of rats exposed to morphine for 10 days: comparison with animals after 20 days of morphine withdrawal. PLoS ONE.

