# Supplementary material for: The influence of rat strain on the development of neuropathic pain and comorbid anxio-depressive behaviour after nerve injury
Source: Sci Rep. 2020 Dec 1;10:20981. doi: 10.1038/s41598-020-77640-8 (PMC7708988; doi:10.1038/s41598-020-77640-8)
Supplement: Supplementary file 1 — Supplementary Information. [file 41598_2020_77640_MOESM1_ESM.docx]

# **Supplementary information**

# **The influence of rat strain on the development of neuropathic pain and comorbid anxio-depressive behaviour after peripheral nerve injury**

S. Hestehave^1,2,3*^, K. SP. Abelson^2^, T. Brønnum Pedersen^1^, D.P. Finn^4^, D.R. Andersson^1^, G. Munro ^5^

^1^; Dept. of Systems Biology, H. Lundbeck A/S, Valby, Denmark.

^2^; Dept. of Experimental Medicine, Faculty of Health and Medical Sciences, University of Copenhagen, Denmark.

^3^; Dept. of Cell & Developmental Biology, University College London, United Kingdom.

^4^: Pharmacology and Therapeutics, School of Medicine, Centre for Pain Research and Galway Neuroscience Centre, NCBES, National University of Ireland Galway, Ireland.

^5^; Hoba Therapeutics, Copenhagen, Denmark.

**Supplementary Figure S1.** The presence of hindpaw contralateral hypersensitivity in SNI rats is strain-dependent. (**A**) 50% mechanical response threshold for the contra-lateral paw on day 182 post nerve-injury. Two-way ANOVA showed no effect of surgery but a significant effect of strain (F[4, 75]= 9.36, P<0.0001). (**B**) Ipsi-lateral paw threshold on day 177 expressed as a percentage of the contra-lateral paw threshold at day 182 [(ipsi/contra) * 100%]. The dotted line indicates 100%. A significant effect of surgery (F[1, 75]= 46.68, P<0.0001), and strain*surgery-interaction (F[4, 75]= 2.669, P=0.039) was observed, and therefore post hoc tests was performed between individual strain- and surgical-groups. Difference between Sham vs. SNI, NS = Not Significant =P>0.05, **P<0.01, ***P<0.001, or indicating strain-differences between surgery-specific groups (LEW; #P<0.05), determined by two-way ANOVA and Bonferroni’s post test. Presented as mean ± S.E.M..

**Supplementary Figure S2.** Anxiety-like behaviour in different rat strains is minimally affected by SNI. (**A-E**) show the time spent in the closed arms of the elevated plus maze for each rat strain at baseline and up to Day 186 post SNI. Two-way Repeated Measures ANOVA with Bonferroni’s post-hoc test, indicating significant difference between Sham vs. SNI at the individual time-points; *P<0.05. (**F)** AUC of time spent in closed arms, presented as scatter plot. Two-way ANOVA showed significant effect of strain (F[4, 75]= 42.78, P<0.0001), and Bonferroni’s post hoc test showed that SD spent significantly less time in the closed arms than LEW, F344/Du and F344/ICO (P<0.001), WKY significantly less than LEW, F344/Du and F344/Ico (P<0.001), and F344/Ico significantly less than F344/Du (P<0.01), as detected pairwise combined for the two surgical groups for each strain. Data are presented as mean ± S.E.M..

**Supplementary Figure S3.** Total consumption of 2% sucrose solution is minimally affected for the majority of the rat strains tested. (**A-E**) Sucrose consumption expressed compared to body-weight of the test-subjects in the cage (1-2 pr cage), as an alternative presentation of anhedonia. Notice that this parameter is recorded on cage level, not subject level, and therefore N=5 for SNI-groups, N=4 for Sham groups. Sucrose consumption was measured on two consecutive test-days, where the sucrose bottle was placed in either the “L”=left or “R”=right side of the cage. Only SD showed significant effects of surgery (F[1,13] = 44.792, P<0.0001). (**F)** AUC of total amount of sucrose consumption related to body-weight. Two-way ANOVA showed significant effect of strain (F[4, 45]= 30.891, P<0.0001), and Bonferroni’s post hoc showed that both the SD- and WKY-strain consumed significantly more than LEW, F344/Ico and F344/Du (P<0.001), and that F344/Du consumed more than LEW (P<0.05), as detected pairwise combined for the two surgical groups for each strain. Data are presented as mean ± S.E.M.

**Supplementary Figure S4:** Bodyweight was minimally affected by induction of neuropathic pain. (**A-E**) Body weight expressed in grams during the study period for each individual strain. **F.** AUC of the body weight curves for the individual animals, presented as scatter plot. Two-way ANOVA detected significant effect of strain: (F[4,75]=137.0, P<0.0001, two-way ANOVA), and Bonferroni’s post hoc test detected that the SD-strain was significantly heavier than all of the other strains (***P<0.001), as detected pairwise combined for the two surgical groups for each strain.. Presented as mean ±SEM, red symbols indicated SNI-groups.

**Supplementary Figure S5:** Sucrose preference test comparing pair- and single-housed SD cages. Sucrose consumption was measured on two consecutive test-days (2 * 24 hours), where the sucrose bottle was placed in either the “L”=left or “R”=right side of the cage, and alternated side following day. Notice that this measure was performed on cage-level, and since three SD-SNI animals were lost during the trial in different cages, three SNI animals were single-housed (n=3 cages), and 4 were pair-housed (n= 2 cages). Sham, n=4. Therefore the group-numbers were too low for the statistical comparisons to give any significant results, but the graphs indicate that there could be an effect of single- vs. co-housing. **(A)** Sucrose-consumption expressed as a percentage of total fluid consumption, used as an index of anhedonia. **(B)** Amount of sucrose consumed compared to body weight in the cage when the test was performed. **(C)** AUC of total amount of sucrose consumption related to body-weight. For the other strains than SD in this graph; SNI, n=5. Presented as mean ± S.E.M.

**Supplementary Figure S6.** Excretion of Fecal Corticosterone Metabolites following nerve injury. **A-E** presents the excretion for individual strains, presented as Mean ±S.E.M. Notice that this parameter is recorded on cage-level, not subject-level, and N=5 for SNI-groups, N=4 for Sham groups. **F.** AUC of Fecal corticosterone metabolites (FCM) over time following SNI or Sham-surgery. Two-way ANOVA showed significant effects of strain followed up by Bonferroni’s post test showed that both the SD- (P<0.05-0.001) and WKY-strain (P<0.01-0.001) had significantly higher FCM-levels than F344/Ico, F344/Du and LEW, as detected pairwise combined for the two surgical groups for each strain.

**Supplementary Figure S7.** Receptor expression, µ opioid receptor (MOP). Presented as scatter plot and mean ±SEM of the signal normalized to beta-actin in the sample. N=8. MOP expression in the RVM **(A)**, PAG **(B)**, PFC **(C)**, and dorsal spinal cords **(D)**. (**E-F**) Representative immunoblots from RVM (**E**) Prefrontal Cortex **(F)** and dorsal spinal cords (**G**), presenting the MOP-bands at ~53kDa, and β-actin at ~42kDa.

**Supplementary Figure S8.** Receptor expression, κ opioid receptor (KOP). Presented as scatter plot and mean ±SEM of the signal normalized to beta-actin in the sample. N=8. KOP expression in the PFC **(A)** and dorsal spinal cords **(B)**. **(C)** Representative immunoblot of samples from the PFC, presenting the KOP-bands at ~43kDa, and β-actin at ~42kDa The unspecified band at ~25kDa was apparent after staining with the KOP-antibody, and likely represents degraded forms of the native KOP.
